# Tumor Cells and the Extracellular Matrix Dictate the Pro-Tumoral Profile of Macrophages in CRC

**DOI:** 10.3390/cancers13205199

**Published:** 2021-10-16

**Authors:** Sara Coletta, Silvia Lonardi, Francesca Sensi, Edoardo D’Angelo, Matteo Fassan, Salvatore Pucciarelli, Arianna Valzelli, Andrea Biccari, William Vermi, Chiara Della Bella, Annica Barizza, Mario Milco D’Elios, Marina de Bernard, Marco Agostini, Gaia Codolo

**Affiliations:** 1 Department of Biology, University of Padova, 35131 Padova, Italy; sara.coletta.1@studenti.unipd.it (S.C.); annica.barizza@studenti.unipd.it (A.B.); marina.debernard@unipd.it (M.d.B.); 2 Section of Pathology, Department of Molecular and Translational Medicine, University of Brescia, 25123 Brescia, Italy; silvia.lona@gmail.com (S.L.); ariannavalzelli00@gmail.com (A.V.); william.vermi@unibs.it (W.V.); 3 Department of Molecular Sciences and Nanosystems, Cà Foscari University of Venice, 30172 Venice, Italy; sensifrancy@gmail.com; 4 Pediatric Research Institute, 35127 Padova, Italy; 5 Department of Surgical, Oncological and Gastroenterological Sciences, University of Padova, 35124 Padova, Italy; edoardo.dangelo@unipd.it (E.D.); salvatore.pucciarelli@unipd.it (S.P.); andrea.biccardi@unipd.it (A.B.); 6 LIFELAB Program, Consorzio per la Ricerca Sanitaria-CORIS, Veneto Region, 35128 Padova, Italy; 7 Department of Medicine, Surgical Pathology Unit, University of Padova, 35124 Padova, Italy; matteo.fassan@unipd.it; 8 Veneto Institute of Oncology, IOV-IRCCS, 35100 Padova, Italy; 9 Department of Experimental and Clinical Medicine, University of Firenze, 50121 Firenze, Italy; chiara.dellabella@unifi.it (C.D.B.); mariomilco.delios@unifi.it (M.M.D.)

**Keywords:** macrophages, colorectal cancer, extracellular matrix, MHC-II, antigen presentation

## Abstract

**Simple Summary:**

The pathogenesis of CRC relies on complex interactions between developing cancer and surrounding tissue, including the immune system. One of the most abundant tumor-infiltrating cell populations is represented by tumor-associated macrophages (TAMs). TAMs play a detrimental role and are associated with a poor prognosis in many tumors and are characterized by an impaired antigen-presenting capability and by immunosuppressive activity. However, their role in CRC is still controversial. Our study aimed to elucidate how the colorectal cancer environment educates macrophages toward a pro-tumoral profile, exploiting them to escape the immune response. We demonstrate that both CRC cells and the extracellular matrix are actively involved in defining the macrophage profile, which is characterized by immunosuppressive activity and an impaired antigen-presenting ability. Dissecting the contribution of the tumor environment to the influence on the macrophage profile will provide additional knowledge for the development of new antitumor strategies.

**Abstract:**

Tumor-associated macrophages (TAMs) are major components of the tumor microenvironment. In colorectal cancer (CRC), a strong infiltration of TAMs is accompanied by a decrease in effector T cells and an increase in the metastatic potential of CRC. We investigated the functional profile of TAMs infiltrating CRC tissue by immunohistochemistry, flow cytometry, ELISA, and qRT-PCR and their involvement in impairing the activation of effector T cells. In CRC biopsies, we evidenced a high percentage of macrophages with low expression of the antigen-presenting complex MHC-II and high expression of CD206. Monocytes co-cultured with tumor cells or a decellularized tumor matrix differentiated toward a pro-tumoral macrophage phenotype characterized by decreased expression of MHC-II and CD86 and increased expression of CD206 and an abundant release of pro-tumoral cytokines and chemokines. We demonstrated that the hampered expression of MHC-II in macrophages is due to the downregulation of the MHC-II transactivator CIITA and that this effect relies on increased expression of miRNAs targeting CIITA. As a result, macrophages become unable to present antigens to CD4 T lymphocytes. Our data suggest that the tumor microenvironment contributes to defining a pro-tumoral profile of macrophages infiltrating CRC tissue with impaired capacity to activate T cell effector functions.

## 1. Introduction

Colorectal cancer (CRC) is the most common malignant cancer of the gastrointestinal tract, and it is considered the second most common cause of death related to cancer. Approximately one third of patients develop metastatic disease [1]. By 2030, the global burden of CRC is expected to reach more than 2.2 million new cases and 1.1 million deaths [1]. Despite significant advances in standard-of-care therapies, patients diagnosed with metastatic CRC achieve a five-year survival rate of 12% [2]. The tumor microenvironment is a complex society of many cell types and the extracellular matrix (ECM), which together generate tumor “tissue.” In this framework, innate immune cells are highly represented, and, among them, the most abundant are tumor-associated macrophages (TAMs) [3,4]. Although macrophages are regarded as involved in antitumor immunity, compelling evidence supports the notion that in the majority of cases, these cells interact with tumor cells to promote the initiation, growth, and metastasis of tumors [5]. Moreover, the remarkable plasticity of macrophages makes them very sensitive to environmental factors, including ECM. In general, macrophages may be divided into two distinct subtypes. M1 macrophages, implicated in initiating and sustaining inflammation, are characterized by the production of proinflammatory mediators and by high surface expression of MHC-II and CD86 molecules and low expression of CD206 [6,7,8]. Conversely, the other subtype, namely, M2 macrophages, triggers an anti-inflammatory response, encourages tissue repair [9], and is characterized by an opposite expression of the aforementioned surface markers [6,7,8]. The tumor environment is thought to educate TAMs toward an M2 phenotype, but the mechanisms underlying this phenomenon are not fully understood [10]. It is now commonly accepted that high numbers of TAMs with an M2-like anti-inflammatory phenotype are typically associated with a poor outcome, whereas polarization toward an M1-like proinflammatory phenotype tends to correlate with a favorable prognosis and longer survival [11]. In most studies concerning CRC, the phenotype of macrophages has been defined on the basis of the general macrophage marker CD68 [12] or, at the very best, considering single polarization markers to identify M1- and M2-like populations [13,14], providing a limited view of macrophage phenotypic diversity. Therefore, the functional profile of macrophages infiltrating CRC remains to be defined. Moreover, although there are studies showing that TAMs may play a protective role in CRC [13,15,16], there are also studies supporting a pro-tumoral role for TAMs and showing that a high macrophage count and M2/M1 ratio in the total tumor area are indicators of a bad prognosis [17,18,19,20,21]. A productive antitumor response requires the contribution of an effective antigen presentation by antigen-presenting cells (APCs) to CD4 T lymphocytes via MHC-II; following activation, CD4 T lymphocytes can activate a cytotoxic T response or can directly carry out a cytotoxic action toward tumor cells in case the latter expose MHC-II–cancer epitope complexes on the surface [22]. A defective presentation of tumor antigens by cancer cells and by TAMs negatively impacts on a productive antitumor response [23,24]. MHC-II antigens, not detectable in normal mucosa, are not expressed by epithelial cells in approximately 58% of CRC. A lack of MHC-II expression is associated with a decrease in tumor-infiltrating T cells and an increase in the metastatic potential of CRC [25]. The idea that a robust adaptive immune response in the tumor microenvironment can counteract CRC progression toward systemic dissemination, thus positively affecting patient outcomes, finds its validation in the recent international acceptance of a consensus immunoscore. This prognostic marker is based on the density of lymphocyte populations, in both the core and invasive margin of the tumor (stage II/III), and establishes the risk of recurrence in patients with colon cancer. A higher infiltration of T cells is associated with a favorable prognosis and longer disease-free survival. Conversely, reduced T lymphocyte infiltration within the tumor is considered unfavorable [26]. T cell activation and proliferation is strictly associated with antigen presentation by macrophages; however, despite the negative association between MHC-II expression in CRC and overall survival [27,28], the contribution of macrophages to antigen presentation failure in CRC is an aspect far to be fully elucidated.

In this work, we investigated the functional profile of macrophages induced by tumor cells and ECM and their ability to activate a T cell-mediated response. We reveal that CRC biopsies are rich in macrophages with low expression of MHC-II and high expression of CD206. Moreover, we demonstrate that monocytes co-cultured with tumor cells or the decellularized tumor matrix (D-ECM) differentiate toward a pro-tumoral macrophage phenotype with low expression of MHC-II and CD86 and strong expression of CD206. These macrophages release pro-tumoral cytokines and chemokines and, most importantly, show an impaired ability to activate T lymphocytes. Finally, we find that reduced expression of MHC-II relies on impaired expression of the MHC-II master regulator CIITA due to the upregulation of miR146b and let-7i.

Overall, our data provide evidence that both tumor cells and the tumor ECM contribute to a pro-tumoral M2-like profile of macrophages in CRC. Moreover, we show that such macrophages are less able to present antigens to T lymphocytes that, in turn, do not efficiently proliferate. This effect could explain, at least in part, the reduced number of T lymphocytes in CRC.

## 2. Materials and Methods

### 2.1. Ethics Statement

This investigation was conducted following ethical standards, the Declaration of Helsinki, and national and international guidelines. Written informed consent was obtained from the individual patients, and the ethics committees of the relevant institutions approved the protocol (Azienda Ospedaliera di Padova Ethical Committee Approved Protocol Number: P448).

The peripheral blood mononuclear cells utilized in this study were derived from buffy coats obtained from healthy blood donors and anonymously provided by the Transfusion Centre of the Hospital of Padova. Written informed consent for the use of the buffy coats for research purposes was obtained from blood donors by the Transfusion Centre. Data related to human samples were all analyzed anonymously. Human leukocytes, provided by the Transfusion Centre of the Hospital of Padova, were obtained not consequently to experimentation on human beings, but as a consequence of voluntary and informed blood donation for transfusions; no approval of an ethics committee is needed in such cases in our institution.

### 2.2. Patients

Both healthy colon (HC) and primary colorectal cancer (CRC) mucosa were collected from eight patients who underwent curative-intent surgery between January 2016 and July 2019 at the First Surgery Clinic (Department of Surgery, Oncology and Gastroenterology, University of Padua). Written informed consent was obtained and the protocol was approved by the ethics committee of the institution (Ethical Committee Approved Protocol Number: 448/2002). All of the patients enrolled fulfilled the following inclusion criteria: histologically confirmed primary adenocarcinoma of the colon, age >18 years, and provision of written informed consent. Patients with a known history of hereditary colorectal cancer syndrome and patients who had undergone neoadjuvant treatments were excluded. The patients’ characteristics are detailed in Table 1.

### 2.3. Preparation of Decellularized Matrices

CRC tissue was obtained from the edge of the infiltrating neoplasia. Healthy colon specimens encompassed the luminal surface, mucosa, and submucosa and were obtained more than 10 cm away from the primary CRC. All surgically collected specimens were kept in cold and sterile phosphate-buffered saline (PBS) for no longer than 2 h before processing. Tissue decellularization, using a detergent-enzymatic treatment, was performed as described in [29]. After decellularization, samples were sterilized with incubation in peracetic acid 0.1% in ethanol 4% for 30 min at room temperature, washed with PBS 1X for 5 days, and finally kept at −80 °C degrees until used or immediately used for further experiments.

### 2.4. Cell Cultures

#### 2.4.1. Tumor and Normal Intestinal Cells

Tumor intestinal epithelial cells HCT-116 (ATCC^®^ CCL-247™) and HT-29 (ATCC^®^ HTB-38™), derived from human colorectal adenocarcinoma, were cultured in DMEM 10% FBS, 10 mM Hepes, 100 U/mL of penicillin, and 100 µg/mL of streptomycin (Sigma, Darmstadt, Germany). Tumor intestinal epithelial cells DLD1 (ATCC^®^ CCL-221™), derived from human colorectal adenocarcinoma, were cultured in RPMI1640 10% FBS, 10 mM Hepes, 100 U/mL of penicillin, and of 100 µg/mL streptomycin. Normal human intestinal cells CCD841 CoN (ATCC^®^ CRL-1790™) were cultured in EMEM 10% FBS, 10 mM Hepes, 100 U/mL of penicillin, and 100 µg/mL of streptomycin. Cells were cultured at 37 °C in a humidified incubator.

#### 2.4.2. Conditioned Media Preparation

For the preparation of HCT-116-, HT-29-, DLD1-, and CCD841-derived conditioned media, 0.5 × 10^6^ cells were seeded onto a six-well plate in 3 mL of cell-specific medium until confluence. The medium was then replaced with 3 mL of DMEM 10% human serum (HS; stimulation medium), 4 mM Hepes, and 50 µg/mL of gentamycin. After 3 days, the medium was collected, filtered through a 0.22 µm filter, frozen in liquid nitrogen, and stored at −80 °C [30].

For the preparation of conditioned media derived from normal and CRC D-ECM, decellularized matrices were incubated in 1 mL of stimulation medium; after 3 days, the medium was collected, filtered through a 0.22 µm filter, frozen in liquid nitrogen, and stored at −80 °C.

#### 2.4.3. Monocyte Isolation

The monocytes derived from buffy coats were prepared as described previously [31].

#### 2.4.4. Co-Culture of Monocytes and Intestinal Cells

A total of 2 × 10^6^ monocytes were seeded onto the bottom chamber of a 24-well plate transwell system for 18 h in 500 µL of RPMI 10% FBS, 4 mM Hepes, and 50 µg/mL of gentamycin. The next day, the culture medium was changed with stimulation medium. A total of 9 × 10^3^ HCT-116, HT-29, DLD1, or CCD841 were seeded, in DMEM 10% human serum (HS), 4 mM Hepes, and 50 µg/mL of gentamycin, onto the upper chamber of the transwell system with a 0.4 µm pore size filter.

Where indicated, monocytes were treated for 5 days with 15% HCT-116-, HT-29-, DLD1-, or CCD841-derived conditioned medium.

After 5 days, the monocytes were harvested and analyzed by flow cytometry and qRT-PCR.

Control monocytes incubated for 5 days in complete medium were not included in the data analysis because, as expected, after 5 d, the cells had died (Supplementary Figure S1).

#### 2.4.5. Co-Culture of Monocytes and Decellularized Matrices

A total of 35 × 10^6^ monocytes were seeded in 100 mm Petri dishes in RPMI 10% FBS, 4 mM Hepes, and 50 µg/mL of gentamycin. After 18 h, cells were harvested and 1.5 × 10^6^ were seeded onto a 24-well plate in the presence of normal or tumor decellularized matrices. After 5 days, the monocytes were collected and analyzed by flow cytometry and qRT-PCR.

As stated above, control monocytes incubated in complete medium were not included.

### 2.5. Immunohistochemistry

Four-micron-thick sections of CRC and their matched normal mucosa were sequentially immunostained as described previously [32]. Briefly, HLA-DR, -DP, -DQ, and -DX/MHC-II (clone V1030, dilution 1:500; Biomeda, Foster City, CA, USA), CD163 (clone 10D6, dilution 1:80; Thermo Scientific, Fremont, CA, USA), and CD3 (clone LN10, dilution 1:100, Leica Biosystems, Wetzlar, Germany) were revealed using Novolink Polymer (Leica Biosystems), followed by a nonpermanent chromogen (AEC). After digitalization using Aperio Scanscope CS (Leica Microsystems), slides were decolored using ethanol (30 min) and the previous antibody was stripped using a 2-mercaptoethanol/SDS solution in a water bath preheated at 56 °C for 30 min. After a 1 h wash in Tris-HCl, sections were unmasked by microwaving in EDTA buffer pH 8.0 and subjected to single staining using CIITA (clone 7-1H, dilution 1:50; Santa Cruz Biotechnology, Santa Cruz, CA, USA) or double immunohistochemistry using Mannose Receptor/CD206 (rb polyclonal, dilution 1:3000; Abcam, Newcastle upon Tyne, UK) coupled with CD163 and CD163 coupled with MHC-II. The first reaction was revealed using Novolink Polymer (Leica Biosystems) followed by DAB, and the second was revealed using Mach4AP Polymer (Biocare Medical, Concord, CA, USA) and Ferangi Blue as chromogen. Slides were counterstained, coverslipped, and digitalized. Then, the two digital slides were synchronized using ImageScope and pictures of the same area were taken to highlight the same cells sequentially stained for MHC-II/CIITA and CD163 or CD163/CD206. In detail, 10 images were taken from digitalized sections of CRC (*n* = 8) and five images from normal colons (*n* = 8). The images covered a total of 0.8 mm^2^ of tissue for each CRC case and 0.4 mm^2^ for each normal mucosa case. In CRC, images were captured from areas with evident macrophage infiltration, including areas in the center of the tumor and invasive margin. Neoplastic cells were always present in at least 10% of the image. For mucinous CRC, images were taken in solid areas where macrophage infiltration was easily measurable. Cells were quantified by optical count using the ImageScope count tool.

### 2.6. Flow Cytometry

Cells were harvested from culture plates using 5 mM Na-EDTA in PBS pH 7.5 and incubated for 15 min at RT with 10% HS in FACS buffer (PBS, 1% BSA) to saturate Fc receptors. Cells were stained with combinations of the following antibodies: BV421-conjugated anti-CD14 (clone 61D3; Ebiosciences, San Diego, CA, USA), PE-conjugated anti-CD68 (clone Y1/82A; BD Biosciences, San Jose, CA, USA), APC-conjugated anti-HLA-DR (clone L243; Ebiosciences), PECyanine7-conjugated anti-CD86 (clone B7-2; Ebiosciences), BB515-conjugated anti-CD206 (clone 19.2; BioLegend, San Diego, CA, USA), and PerCP-Cyanine5.5-conjugated anti-CD163 (clone GHI/61; Ebiosciences). The fixable cell viability dye eFluor780 (Ebiosciences) was used to exclude dead cells from the analysis. For intracellular staining of CD68, cells were fixed with 3.7% formaldehyde and permeabilized with a PermWash solution (1% FBS and 0.2% saponin in PBS). Cells were resuspended in FACS buffer and analyzed by BD LSFortessa X20 (Becton Dickinson, Franklin Lakes, NJ, USA). Values were expressed as *n*-fold change in the median fluorescence intensity (MFI) of monocytes incubated with tumor cells or tumor decellularized matrices versus control cells. Data were analyzed using FlowJo version 10.3 (Tree Star, Ashland, OR, USA).

### 2.7. RNA Extraction

Total RNA was extracted with TRIzol reagent (Thermo Fisher Scientific, Waltham, MA, USA) according to the manufacturer’s protocol. RNA was quantified using a NanoDrop 1000 spectrophotometer (Nanodrop). The RNA integrity and content of miRNAs in each sample were assessed by capillary electrophoresis using the Agilent Bioanalyzer 2100 with the RNA 6000 Nano and the Small RNA Nano LabChips, respectively (Agilent Technologies). Only samples with an RNA integrity >7 and with a concentration of small RNAs <30% were used for miRNAs or mRNA gene expression analysis.

### 2.8. qRT-PCR

#### 2.8.1. mRNAs

One microgram of total RNA was retrotranscribed using a High-Capacity cDNA Reverse Transcription Kit (Thermo Fisher Scientific). Retrotranscription was performed at 37 °C for 2 h following the manufacturer’s instructions. After precipitation, 5 ng of cDNA were used in qRT-PCR performed in a QuantStudio5 Real-Time PCR System (Thermo Fisher Scientific). qRT-PCR was performed in 10 µL of SYBR Green master mix (Thermo Fisher Scientific) according to the following cycle: 95 °C for 5 min; 95 °C for 15 s, and 60 °C for 1 min for 40 cycles. Experiments were performed with at least three technical replicates. For each sample, data were normalized to the endogenous reference gene β-actin. The primers used were as follows: β-actin forward, 5′-TGAGATGCGTTGTTACAGGA-3′; reverse, 5′-ACGAAAGCAATGCTATCA-3′; CIITA forward, 5′-GGTCCAGGGTTTGAGTTCAT-3′; reverse, 5′-TGATTTGGGGTGGCTTGTTA-3′.

#### 2.8.2. miRNAs

miR146b-5p (Thermo Fisher Scientific—Assay ID 001097) and let-7i-5p (Thermo Fisher Scientific—Assay ID 002221) expression was determined using TaqMan^®^ MicroRNA Assays (Thermo Fisher Scientific) as reported elsewhere [33] Retrotranscription of each specific miRNA was accomplished using a TaqMan^®^ MicroRNA Reverse Transcription Kit with 10 ng of total RNA (Thermo Fisher Scientific) following the manufacturer’s instructions. RT-PCR was then performed using TaqMan^®^ Universal PCR Master Mix II, no UNG (Thermo Fisher Scientific), and the specific primers from the TaqMan^®^ MicroRNA Assay. At least three technical replicates were performed for each reaction. miRNAs expression was normalized to the U6 small nuclear RNA (U6 snRNA; Thermo Fisher Scientific—Assay ID 001973). Reactions were run in 96 CFX (BioRad) with the following program: 95 °C for 10 min, 35 cycles of 95 °C for 15 s, and 60 °C for 1 min. Data analysis was carried out according to the ΔΔCt method for both mRNAs and miRNAs.

### 2.9. Quantification of Cytokines, Chemokines, and Hyaluronic Acid (HA) in Culture Supernatants

Culture supernatants of monocytes/macrophages co-cultured with tumor cells, conditioned media, and decellularized matrices, as well as the tumor cells’ conditioned media, were collected and stored at −80 °C for subsequent quantification of the cytokine content.

#### 2.9.1. ELISA

An ELISA kit specific for TGF-β (eBiosciences) was used following the manufacturer’s instructions.

#### 2.9.2. Multiplex

Quantification of IL-6, IL-10, CCL17, CCL18, and CCL22 was performed using a specific multiparametric detection kit (Milliplex MAP; Millipore).

#### 2.9.3. HA Quantification

The amount of HA in the culture supernatants and in the IEC and HCT116 conditioned media was quantified using an ELISA kit (Echelon Biosciences, Salt Lake City, UT, USA).

### 2.10. T Cell Proliferation Assay

Peripheral blood mononuclear cells (PBMCs) isolated from two tetanus toxoid (TT)-vaccinated subjects were seeded at 1.5 × 10^5^/well density on 96-well round-bottom plates. Cells were incubated in DMEM 10% HS, 0.5 µg/mL of TT in the presence of 15% HCT-116 or CCD841 conditioned medium, or in the presence of 25% of culture supernatants derived from a five-day co-culture of monocytes together with tumor or normal D-ECM.

After four days of stimulation, 1 µCi/well ^3^[H]-TdR was added and the radionuclide uptake was measured after 18 h, as reported in [34].

Data were expressed as *n*-fold proliferating T cells in response to TT in PBMCs cultured in the presence of 15% HCT-116 versus CCD841 conditioned medium or in the presence of 25% of culture supernatants derived from a five-day co-culture of monocytes together with tumor versus normal D-ECM.

### 2.11. Statistics

Data are reported as the mean ± SEM. Student’s *t*-test and Mann–Whitney *U*-test were used for statistical analysis of the differences between experimental groups. A *p*-value ≤ 0.05 was considered significant.

## 3. Results

### 3.1. Macrophages Expressing Low MHC-II and High CD206 Infiltrate CRC Mucosa

**Figure 1:**
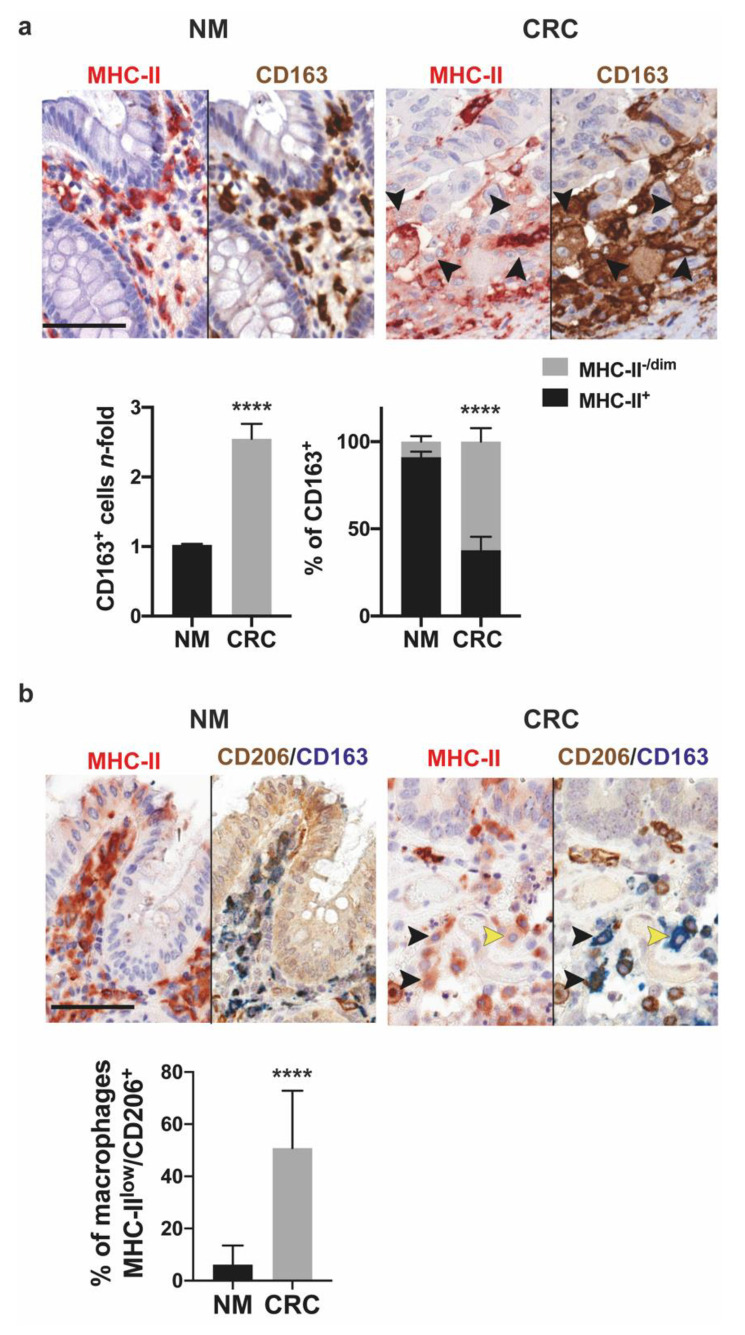
Profile of macrophages infiltrating colorectal human cancer. (**a**) Increased infiltration of macrophages showing low or no expression of MHC-II in tumor tissue. Immunohistochemical sequential staining for MHC-II (red) and CD163 (brown) was performed on the same FFPE section of normal mucosa (NM) and colorectal tumor (CRC) biopsy, respectively. Arrows highlight CD163^+^ macrophages with low or no expression of MHC-II (–/dim). The number of CD163^+^ macrophages in eight patients was calculated and expressed as *n*-fold mean values ± SEM vs. normal mucosa (bottom, left plot); the right bottom plot shows the percentage of macrophages with high (+) or no/low (–/dim) expression of MHC-II in normal and CRC tissue expressed as mean ± SEM. Scale bar, 20.6 µm. (**b**) MHC-II-negative macrophages highly express CD206 in CRC tissue. Representative images from NM and CRC samples sequentially immunostained for MHC-II (red) and CD206 (brown)/CD163 (blue). Black arrows highlight CD163+CD206+MHC-II-/dim macrophages and yellow arrows CD163+CD206-MHC-II-/dim. Images are snapshots from digitally scanned slides (digital magnification 400×). Data are expressed as mean ± SEM. Scale bar, 66 µm. Significance was determined by Student’s *t*-test: **** *p* < 0.0001.

In order to determine whether macrophages infiltrating CRC tissue display anti-inflammatory/pro-tumoral features, we performed immunohistochemical analysis of human intestinal biopsies from eight cases of CRC and their matched controls. We found that CRC tissue was more densely infiltrated with CD163^+^ macrophages than normal matched intestinal mucosa. Moreover, the large majority of CD163^+^ macrophages in normal mucosa showed a robust expression of MHC-II. On the contrary, a significant percentage of tumor-associated macrophages in CRC samples was characterized by weak or absent expression of MHC-II (Figure 1a).

It is interesting that most macrophages with low or negative expression of MHC-II in CRC were characterized by high expression of CD206 (Figure 1b). These results are consistent with the notion that the macrophages infiltrating CRC exhibited an M2-like immunosuppressive profile.

Notably, peri-tumor areas with important infiltration of MHC-II^-/dim^ macrophages were characterized by a low number of CD3^+^ T cells (Supplementary Figure S2a,c), while in areas with massive CD3^+^ infiltration macrophages with a robust expression of MHC-II were abundant (Supplementary Figure S2b,d).

### 3.2. Tumor Cells Educate Monocytes to Acquire a Pro-Tumoral Macrophage-like Profile

Macrophages are very dynamic and plastic cells, as they display a broad spectrum of activation states with distinctive phenotypes and functions according to the microenvironment [7]. To dissect the players in the tumor microenvironment involved in the induction of immunosuppressive macrophages, we evaluated in vitro the contribution of tumor cells to macrophage differentiation and polarization.

**Figure 2:**
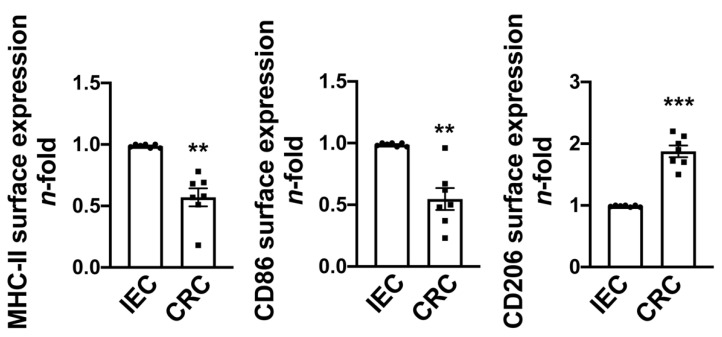
Profile of monocyte-differentiated macrophages co-cultured with tumor cells. Monocytes from healthy donors were plated on the bottom chamber of a transwell system and co-cultured with normal human intestinal epithelial cells (IECs) or HCT116 colon cancer cells (CRCs) plated onto the upper chamber. After five days, cells were harvested from the bottom chamber and analyzed by flow cytometry for the expression of MHC-II, CD86, and CD206 gated on CD68^+^ cells. Data are expressed as *n*-fold vs. IEC ± SEM of seven independent experiments from seven different donors. Significance was determined by Student’s *t*-test: ** *p* < 0.01 and *** *p* < 0.001.

Because tumor-infiltrating macrophages are derived, at least in part, from newly recruited monocytes, we co-cultured human monocytes freshly isolated from buffy coats with human colorectal cancer (CRC) cells and normal human intestinal epithelial cells (IEC). To determine the cell profile, in addition to the monocyte lineage marker CD14 and the macrophage lineage marker CD68, we assessed the expression of markers specific for proinflammatory macrophages, such as MHC-II and CD86, as well as the expression of CD206, which is upregulated in macrophages exhibiting anti-inflammatory activity. After a five-day co-culture, monocytes differentiated to macrophages, as evidenced by the decreased expression of CD14 paralleled by the increased expression of CD68. Note that this occurred regardless of whether cells co-cultured with monocytes were tumoral or not (Supplementary Figure S3). Because CD68, a glycoprotein known as a myeloid-specific marker, is abundantly expressed by macrophages [32], we decided to perform all the other flow cytometry analyses on CD68^+^ gated cells. It is interesting that the presence of CRC cells decreased the surface expression of MHC-II and CD86 in macrophages compared to monocytes exposed to IEC. This was paralleled by higher expression of CD206 (Figure 2) (for the detailed gating strategy and median fluorescence intensity values, see Supplementary Figure S3 and Table S1. An analogous effect on monocyte differentiation toward an immunosuppressive pro-tumoral profile was observed in monocytes co-cultured with two other different CRC cell lines (HT-29 and DLD1 cells), as shown in Supplementary Figure S4. These data suggest that in the presence of tumor cells, monocytes acquire a macrophage-like phenotype with an anti-inflammatory/pro-tumoral profile.

### 3.3. Tumor Cells Induce the Production of Anti-Inflammatory and Pro-Tumoral Cytokines and Chemokines in Monocyte-Differentiated Macrophages

**Figure 3:**
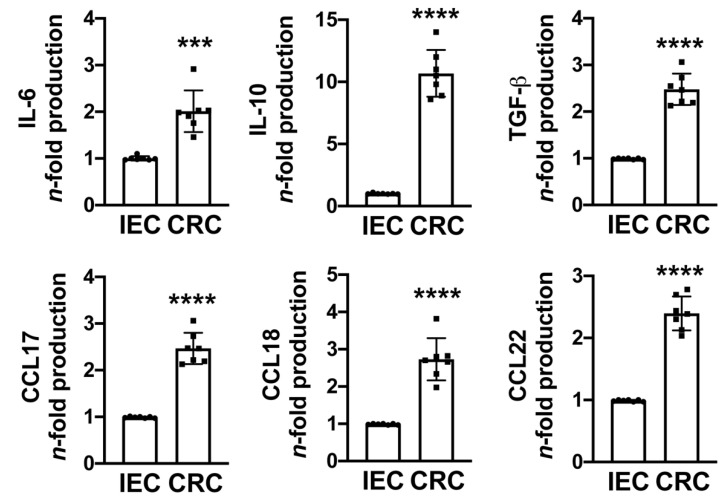
Cytokine and chemokine production by monocyte-differentiated macrophages co-cultured with tumor cells. Monocytes from healthy donors were plated on the bottom chamber of a transwell system and co-cultured with normal human intestinal epithelial cells (IECs) or HCT116 colon cancer cells (CRCs) plated onto the upper chamber. After 5 days, culture supernatants were harvested and the IL-6, IL-10, TGF-β, CCL17, CCL18, and CCL22 released was quantified by ELISA. Data are expressed as *n*-fold vs. IEC ± SEM of seven independent experiments from seven different donors. Significance was determined by Student’s *t*-test: *** *p* < 0.001 and **** *p* < 0.0001.

The functional profile of macrophages is not just defined by surface markers. Tumor macrophages also secrete a plethora of immunosuppressive cytokines, chemokines, and enzymes that facilitate the immune escape of the tumor [35,36]. We found that monocytes exposed to CRC cells secreted abundant amounts of IL-6, IL-10, and TGF-β, as well as pro-tumoral chemokines such as CCL17, CCL18, and CCL22 (Figure 3).

It is interesting that we obtained essentially identical results by incubating monocytes with a medium containing 15% of the conditioned media from a three-day confluent culture of CRC cells (Supplementary Figure S5), which suggests that the cytokines and chemokines detected in the co-cultures are mainly produced by monocyte-differentiated macrophages. This is also supported by the evidence that neither cytokines nor chemokines are present in the tumor cells conditioned medium; the only exception concerns TGF-β, which was also produced, as expected, by tumor cells (Supplementary Figure S6). Taken together, our findings identify colorectal cancer cells as a crucial source of factors enabling monocytes to acquire a pro-tumoral macrophage-like profile.

### 3.4. The Extracellular Matrix Educates Monocytes to Acquire a Pro-Tumoral Macrophage-like Profile

In the past two decades, not only have tumor cells been described to be relevant for cancer progression as far as determining the behavior of cells populating the tumor tissue, including macrophages, but so has the ECM [37,38,39].

Recently, tissue decellularization has emerged as an alternative tool for unraveling the complex role of the ECM in tumor progression [40,41,42,43].

**Figure 4:**
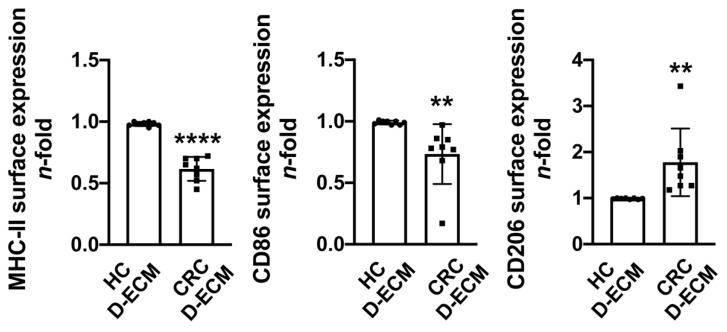
Profile of monocyte-differentiated macrophages co-cultured with decellularized matrices. Monocytes were isolated from healthy donors and co-cultured with a matched normal decellularized matrix (HC D-ECM) or a tumor decellularized matrix (CRC D-ECM) for five days. Cells were harvested and analyzed by flow cytometry for the expression of MHC-II, CD86, and CD206 gated on CD68^+^ cells. Data are expressed as *n*-fold vs. the HC D-ECM of each patient ± SD of eight patients. Significance was determined by Student’s *t*-test: ** *p* < 0.01 and **** *p* < 0.0001.

**Figure 5:**
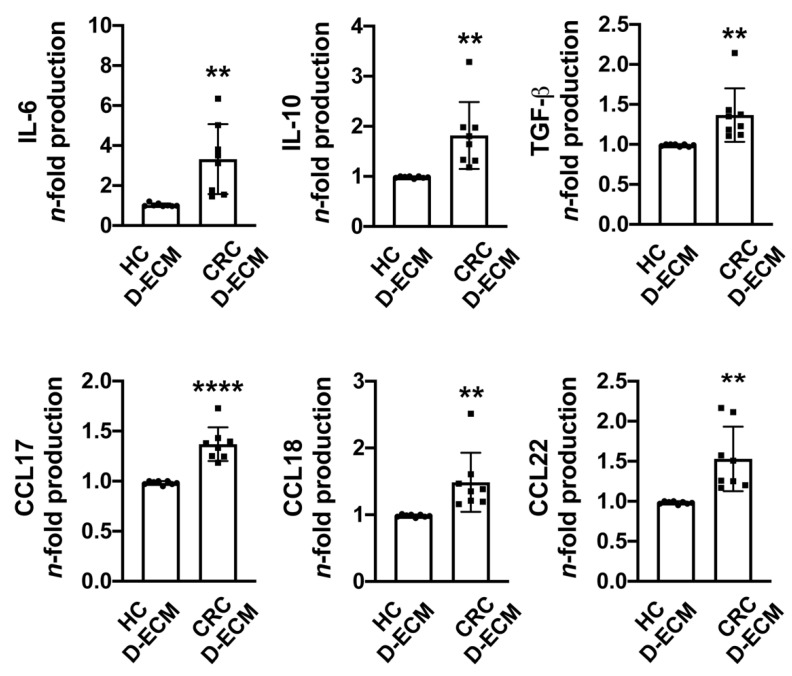
Cytokine and chemokine production by monocyte-differentiated macrophages following exposure to the D-ECM. Monocytes were isolated from healthy donors and co-cultured with a matched normal decellularized matrix (HC D-ECM) or a tumor decellularized matrix (CRC D-ECM). After five days, culture supernatants were harvested and the IL-6, IL-10, TGF-β, CCL17, CCL18, and CCL22 released were quantified by ELISA. Data are expressed as *n*-fold vs. the HC D-ECM of each patient ± SD of eight patients. Significance was determined by Student’s *t*-test: ** *p* < 0.01 and **** *p* < 0.0001.

To evaluate the contribution of the tumor ECM to modulating monocyte differentiation, we co-cultured human monocytes with the decellularized matrix (D-ECM) from normal intestinal mucosa (HC) and from the mucosa of colorectal cancer (CRC) patients. As above, the expression of surface markers and the production of cytokines and chemokine were determined by flow cytometry (Figure 4) and ELISA (Figure 5), respectively (for the median fluorescence intensity values and ELISA raw data, see Supplementary Tables S1 and S2). The results revealed that after five days, monocytes acquired a macrophage-like profile, as demonstrated by the decreased expression of CD14 and the increased expression of CD68, and this occurred regardless of the source of the D-ECM (data not shown). Conversely, only D-ECM from CRC downregulated the expression of MHC-II and CD86, both markers of proinflammatory macrophages, in monocytes, whereas it upregulated the expression of CD206, a marker used for distinguishing anti-inflammatory/pro-tumoral macrophages from proinflammatory ones (Figure 4).

It is interesting that the conditioned media from the normal and tumor D-ECMs did not induce monocyte differentiation toward a macrophage-like profile: the expression of CD14 and that of CD68 remained unaltered with respect to freshly isolated monocytes (data not shown), and no effect on MHC-II expression was observed (Supplementary Figure S7). Therefore, unlike tumor cells that dictate the profile of monocyte-derived macrophages via soluble factors, in the case of the ECM, monocytes must be in close contact with it to differentiate toward cells with a pro-tumoral profile.

To supplement this analysis, the levels of IL-6, IL-10, TGF-β, CCL17, CCL18, and CCL22 were determined in the conditioned media derived from the co-cultures. The production of all cytokines and chemokines analyzed increased significantly in monocyte-differentiated macrophages co-cultured with the tumor D-ECM compared to their normal counterparts (Figure 5), which suggests that the differentiated macrophages are immunosuppressive and thus expected to favor tumor progression. In order to exclude the possibility that the polarization effect of the CRC D-ECM on macrophages could be related to the microsatellite instability of native CRC biopsy, we cross-referenced the data with the immunophenotypic characterization of the mismatch–repair proteins that is routinely performed at our institution, showing a microsatellite stability profile in six out of eight patients, as detailed in Table 1.

### 3.5. The Down-Modulation of MHC-II Involves the Targeting of CIITA by miR146b and let-7i

**Figure 6:**
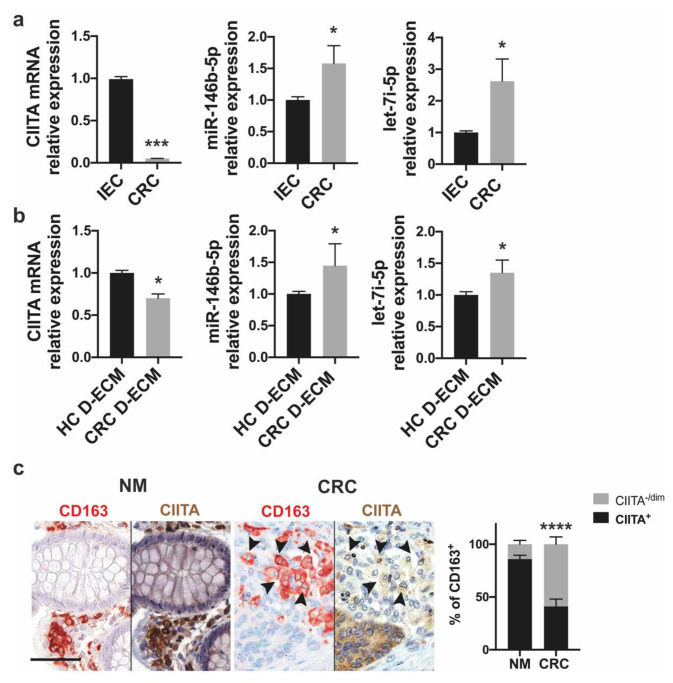
Expression of CIITA and miR-146b-5p and let-7i-5p in monocyte-derived macrophages. (**a**) Monocytes from healthy donors were seeded on the bottom chamber of a transwell system and co-cultured with normal human intestinal epithelial cells (IEC; control cells) or HCT116 colon cancer cells (CRC) seeded onto the upper chamber. After five days, cells were harvested from the bottom chamber and the expression of CIITA mRNA was analyzed by qRT-PCR. Data were normalized to the endogenous reference gene β-actin (left panel). The relative expression of miR-146b-5p and let-7i-5p were evaluated and data were normalized to an endogenous reference gene (U6). Values of macrophages cultured with control cells were taken as the reference and set as 1; the expression of macrophages cultured with CRC cells was relative to the expression of control cells (right panel). (**b**) Monocytes were isolated from healthy donors and co-cultured with a matched normal decellularized matrix (HC D-ECM) or a tumor decellularized matrix (CRC D-ECM). After five days, cells were harvested and the expression of CIITA, miR-146b-5p, and let-7i-5p was analyzed by qRT-PCR. Data were normalized to the housekeeping β-actin and U6, respectively. The values of macrophages cultured with the HC D-ECM were taken as the reference and set as 1; the expression of macrophages cultured with the CRC D-ECM was relative to the expression of the controls. Data are expressed as the mean ± SEM of five independent experiments. (**c**) Immunohistochemical sequential staining for CD163 (red) and CIITA (brown) was performed on eight formalin-fixed paraffin-embedded samples of normal mucosa (NM) and colorectal tumor (CRC) biopsy, respectively. Arrows highlight CD163^+^ macrophages with low or no expression of CIITA (–/dim). The right plot shows the percentage of macrophages with high (+) or no/low (–/dim) expression of CIITA in normal and CRC tissue expressed as the mean ± SEM. Scale bar, 66 µm. Significance was determined by Student’s *t*-test: * *p* < 0.05, *** *p* < 0.001, and **** *p* < 0.0001.

The expression of MHC-II molecules is finely tuned by several transcription factors [44], but the major histocompatibility complex Class II Trans Activator (CIITA) is considered the master regulator of transcription [45]. In the absence of CIITA, the expression of genes encoding for MHC-II molecules is prevented [46,47,48]. Based on this notion, we assessed whether the decrease in MHC-II molecules in monocyte-differentiated macrophages might be due to the downregulation of CIITA. In agreement with the decrease in MHC-II induced by tumor cells, after a five-day co-culture, the expression of CIITA was strongly decreased (Figure 6a, left panel, and Supplementary Figure S8a). The expression of CIITA was also significantly reduced in monocyte-differentiated macrophages co-cultured with the D-ECM, even if to a lesser extent (Figure 6b, left panel). Moreover, sequentially immunostained sections of colorectal carcinoma (*n* = 5) and matched normal mucosa showed decreased expression of CIITA in CD163^+^ macrophages compared to normal tissue (Figure 6c). It is known that miRNAs regulate gene expression, leading to translational repression and/or transcript degradation [49]. miR-146b-5p and let-7i-5p have been shown to target CIITA [50]; moreover, the dysregulation of miR-146b-5p has been documented in a variety of malignances, including CRC [51,52,53], and it has been demonstrated that an increased expression of let-7i-5p is associated with CRC metastasis [54]. Moreover, the increased expression of let-7i-5p in TAMs induces an anti-inflammatory pro-tumoral profile [55]. To determine whether the two miRNAs are implicated in antagonizing the expression of CIITA, we evaluated their expression in monocyte-derived macrophages co-cultured with either tumor cells or the tumor D-ECM. As shown in the right panels of Figure 6a,b and in Supplementary Figure S8b, we observed that the expression of miR-146b-5p and let-7i-5p increased under both conditions.

### 3.6. Decreased MHC-II Expression Affects the Activation of a Specific T Cell Response

**Figure 7:**
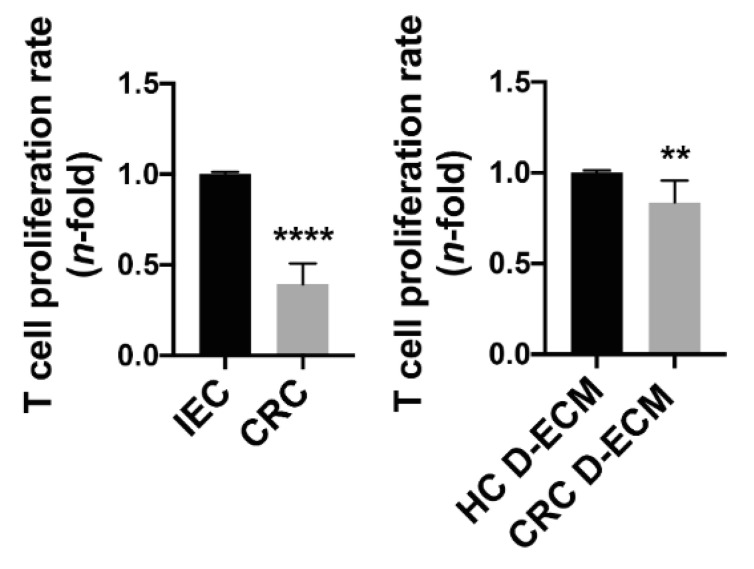
The impact of MHC-II expression on the antigen presentation ability of monocyte-differentiated macrophages to T lymphocytes. PBMCs were isolated from tetanus-vaccinated subjects and stimulated with 15% CCD841 (IEC) conditioned medium and 15% HCT-116 (CRC) conditioned medium (left panel) or stimulated with 25% co-culture medium of monocytes + the HC D-ECM and the monocytes CRC D-ECM (right panel) in the presence of 0.5 µg/mL of tetanus toxoid. Lymphocyte proliferation was measured by ^3^[H]-TdR incorporation. Data are expressed as *n*-fold vs. IEC or vs. the normal D-ECM ± SEM of triplicate samples from two different subjects. Significance was determined by Student’s *t*-test: ** *p* < 0.01 and **** *p* < 0.0001.

Inhibition of T cell activation is among the mechanisms by which TAMs may promote tumor growth [56,57]. Given the notion that a fruitful antitumor response requires the effective presentation of tumor antigens via MHC-II by APCs to CD4 T lymphocytes [22], we evaluated whether the decreased expression of MHC-II molecules in monocyte-derived macrophages exposed to tumor cells or the tumor D-ECM impacts on their ability to present antigens to T cells, affecting the proliferation rate. PBMCs were isolated from tetanus toxoid-vaccinated subjects and incubated with 15% of conditioned medium derived from tumor cells and normal intestinal cells or with 25% of conditioned medium derived from the co-culture of monocytes and the D-ECM, both normal and tumor. As shown in Figure 7, in the presence of conditioned medium from tumor cells, as well as in the presence of conditioned medium from monocytes co-cultured with the tumor D-ECM, T cell proliferation was significantly impaired in comparison to the respective controls. These findings highlight the role of tumor-associated macrophages in participating in tumor progression via the impairment of antigen presentation toward T cells.

## 4. Discussion

In the tumor microenvironment, a complex network of interactions occurs between the cell compartment, immune cells included, and the ECM. These interactions have a tremendous impact on the course of the disease [58]. Establishing an immunosuppressive microenvironment by changing cytokines or reprogramming immune cells guarantees that tumor cells escape immune surveillance, facilitating the initiation and progression of CRC. In CRC, reduced infiltration of T cells correlates with a poor clinical outcome [26]; however, the contribution of macrophages has not been considered. Macrophages are the most abundant immune cells infiltrating tumors and are crucial in activating effector T cells via antigen presentation [22]. The contribution of the antigen presentation capacity of macrophages to unsuccessful T lymphocyte activation and proliferation in CRC has never been explored before.

Herein, we revealed that macrophages infiltrating CRC tissue, identified by the marker CD163 [59], have a pro-tumoral profile. In accordance, monocytes co-cultured with CRC cells or CRC decellularized matrices differentiated into macrophages with an anti-inflammatory/pro-tumoral profile, with an impaired capacity to present antigens and to activate the T cell-mediated immune response. This is in accordance with the evidence that the lack of MHC-II expression correlates with a decrease in tumor-infiltrating T cells and increased metastatic potential [25].

It is now established that not only do tumor cells play a crucial role in regulating the pro-tumoral behavior and function of TAMs, but so does the ECM. Herein, we revealed that tumor cells and the decellularized tumor matrix induce the differentiation of monocytes in macrophages, characterized by high surface expression of CD206 and reduced expression of MHC-II and CD86.

CD206 is a mannose receptor involved in the modulation of multiple cellular activities, but it is often found highly expressed in macrophages infiltrating tumors and is implicated in anti-inflammatory functions [7]. MHC-II is the molecule responsible for antigen presentation toward CD4 T lymphocytes, and CD86 is a costimulatory signal required for full activation of T cells. These two molecules are commonly associated with an M1-like proinflammatory phenotype [60]. Our data suggest that the phenotype acquired by differentiated macrophages resembles the M2-like anti-inflammatory profile that typically characterizes pro-tumoral TAMs.

These data are corroborated by other analyses of the mediators released: in macrophages exposed to tumor cells or the tumor ECM higher levels of IL-6, IL-10, TGF-β, CCL17, CCL18, and CCL22 were detected. All of these are crucial in creating an environment rich in pro-tumoral and anti-inflammatory mediators [36]. Indeed, IL-6 is a cytokine that promotes angiogenesis, proliferation, and migration of tumor cells and contributes to a favorable environment for metastasis [61]. IL-10 and TGF-β are immunosuppressive cytokines that facilitate the differentiation of T regulatory cells [62], and the chemokines CCL17, CCL18, and CCL22 secreted by macrophages are responsible for the recruitment of naïve and Th2 lymphocytes, which are ineffective in terms of antitumor responses [35,43].

Dendritic cells are defective in CRC, as well as in many other solid tumors; thus, macrophages represent the most abundant population of APCs [63]. Loss or downregulation of MHC-II expression is considered one of the major strategies used by tumors to evade the immune system [64]. Our data demonstrate that tumor cells and the decellularized matrix both contribute to decreasing the MHC-II expression in macrophages, affecting their antigen presentation capacity, which in turn leads to impaired T cell activation and proliferation. These findings are in accordance with a previous study by Warabi et al. reporting that MHC-II-negative CRC tissue exhibits a lower grade of T cell infiltration, allowing tumors to escape immune surveillance [25].

MHC-II gene expression is finely regulated by the master regulator CIITA, and the lack of or reduced MHC-II expression depends on alteration of the expression of this transactivator [50]. In line with this, we showed that tumor cells and the decellularized matrix modulate the expression of CIITA in differentiated macrophages, corroborated by the in vivo correlate demonstrating reduced expression of CIITA in tumor-infiltrating macrophages. The gene expression of CIITA can be regulated at the post-transcriptional level by miRNAs [50], and both tumor cells and the tumor ECM trigger the upregulation of miR-146b-5p and let-7i-5p, which target the mRNA for CIITA [50]. Note that dysregulation of the two miRNAs has been reported in a variety of malignancies [65], including CRC, in which it has been shown that aberrant high expression of miR-146b-5p, and also let-7i-5p, correlate with advanced tumor stage and metastasis [53,54]. Notably, the increased expression of let-7i-5p in TAMs results in conversion into pro-tumoral macrophages’ phenotype [55]

Overall, our findings point to the crucial role of the tumor microenvironment, including both tumor cells and the tumor ECM, in controlling macrophage polarity toward an immunosuppressive phenotype. In this regard, we can speculate that a common factor should be responsible for such an effect. Hyaluronic acid (HA) is a long-chain polysaccharide and major component of the tumor-associated ECM. Its role in cancer initiation and progression has been established [66,67,68]. HA is overproduced by tumor cells and deposited in the ECM of the tumor microenvironment [69,70,71]. Among others, HA affects the function of immune cells, triggering a pro-tumoral immunosuppressive M2 polarity in tumor-infiltrating macrophages [30,72]. It is interesting that, as already reported [41], decellularized matrices from CRC are enriched in HA compared to normal matched controls. Moreover, culture supernatants of monocytes co-cultured with tumor cells and conditioned medium of tumor cells were both enriched in HA (Supplementary Figure S9).

These observations are suggestive of a contribution of HA to modulating the profile of macrophages infiltrating CRC, although this is an issue that needs to be further investigated.

## 5. Conclusions

The present work highlights the contribution of tumor cells and the ECM to promoting the differentiation of macrophages toward a pro-tumoral anti-inflammatory phenotype. Such cells create an immunosuppressive environment through the release of anti-inflammatory mediators that contribute to facilitating the differentiation of T regulatory cells, inducing ineffective antitumor responses in the tumor microenvironment. Differentiated macrophages also exhibit reduced capacity to activate effector T cells because of an impaired antigen presentation ability; this might be one of the mechanisms accounting, at least in part, for the reduced number of T cells infiltrating tumor tissue.

## Figures and Tables

**Table 1:** Clinicopathological characteristics of CRC patients.

CRC Patients (*n* = 8)		
Age	Median (range), yrs	66 (58–88)
Sex	Male	6 (75%)
	Female	2 (25%)
TNM	I	0 (0%)
	II	5 (62.5)
	III	3 (37.5)
	IV	0 (0%)
Microsatellite status	Stable	6 (75%)
	Unstable	1 (12.5%)
	Not available	1 (12.5%)
Grade	1	0 (0%)
	2	6 (75%)
	3	2 (25%)
	4	0 (0%)
Tumor histotype	Adenocarcinoma	6 (75%)
	Mucinous	2 (25%)

CRC, colorectal cancer; Yrs, years; TNM, pathological Tumor, Node, Metastasis stage.

## Data Availability

Data sharing not applicable.

## Supplementary Materials

The following are available online at https://www.mdpi.com/article/10.3390/cancers13205199/s1. Figure S1: Representative cytograms of untreated monocytes. Figure S2: A higher number of MHC-IIdim/- CD163+ macrophages correlate with a lower number of CD3+ T cells infiltrating tumor areas in CRC. Figure S3: Examples of the flow cytometric gating strategy adopted for data analysis. Figure S4: Profile of monocyte-differentiated macrophages co-cultured with tumor cells. Figure S5: Conditioned medium from colorectal cancer cells promotes monocyte differentiation toward a pro-tumoral macrophage-like profile. Figure S6. Cytokines and chemokines in conditioned media from colorectal cancer cells. Figure S7: Conditioned medium from the D-ECM does not affect MHC-II surface expression. Figure S8. Expression of CIITA, miR-146b-5p and let-7i-5p in monocyte-derived macrophages. Figure S9: HA quantification in monocytes co-cultured with tumor cells and in a HCT116 conditioned medium. Table S1: Meadian Fluorescent Intensity for each donor or patient. Table S2: Cytokines and chemokines concentrations (pg/mL).

## References

[1] Arnold M., Sierra M.S., Laversanne M., Soerjomataram I., Jemal A., Bray F. (2017). Global Patterns and Trends in Colorectal Cancer Incidence and Mortality. Gut.

[2] Siegel R.L., Miller K.D., Fuchs H.E., Jemal A. (2021). Cancer Statistics, 2021. CA Cancer J. Clin..

[3] Mantovani A., Ponzetta A., Inforzato A., Jaillon S. (2019). Innate Immunity, Inflammation and Tumour Progression: Double-Edged Swords. J. Intern. Med..

[4] Qian B.-Z., Pollard J.W. (2010). Macrophage Diversity Enhances Tumor Progression and Metastasis. Cell.

[5] Mantovani A., Sica A., Locati M. (2005). Macrophage Polarization Comes of Age. Immunity.

[6] Sica A., Erreni M., Allavena P., Porta C. (2015). Macrophage Polarization in Pathology. Cell. Mol. Life Sci..

[7] Murray P.J., Allen J.E., Biswas S.K., Fisher E.A., Gilroy D.W., Goerdt S., Gordon S., Hamilton J.A., Ivashkiv L.B., Lawrence T. (2014). Macrophage Activation and Polarization: Nomenclature and Experimental Guidelines. Immunity.

[8] Biswas S.K., Chittezhath M., Shalova I.N., Lim J.-Y. (2012). Macrophage Polarization and Plasticity in Health and Disease. Immunol. Res..

[9] Mantovani A., Sica A., Sozzani S., Allavena P., Vecchi A., Locati M. (2004). The Chemokine System in Diverse Forms of Macrophage Activation and Polarization. Trends Immunol..

[10] Solinas G., Germano G., Mantovani A., Allavena P. (2009). Tumor-Associated Macrophages (TAM) as Major Players of the Cancer-Related Inflammation. J. Leukoc. Biol..

[11] Bindea G., Mlecnik B., Tosolini M., Kirilovsky A., Waldner M., Obenauf A.C., Angell H., Fredriksen T., Lafontaine L., Berger A. (2013). Spatiotemporal Dynamics of Intratumoral Immune Cells Reveal the Immune Landscape in Human Cancer. Immunity.

[12] Chaput N., Svrcek M., Aupérin A., Locher C., Drusch F., Malka D., Taïeb J., Goéré D., Ducreux M., Boige V. (2013). Tumour-Infiltrating CD68+ and CD57+ Cells Predict Patient Outcome in Stage II–III Colorectal Cancer. Br. J. Cancer.

[13] Koelzer V.H., Canonica K., Dawson H., Sokol L., Karamitopoulou-Diamantis E., Lugli A., Zlobec I. (2016). Phenotyping of Tumor-Associated Macrophages in Colorectal Cancer: Impact on Single Cell Invasion (Tumor Budding) and Clinicopathological Outcome. OncoImmunology.

[14] Ålgars A., Irjala H., Vaittinen S., Huhtinen H., Sundström J., Salmi M., Ristamäki R., Jalkanen S. (2012). Type and Location of Tumor-Infiltrating Macrophages and Lymphatic Vessels Predict Survival of Colorectal Cancer Patients. Int. J. Cancer.

[15] Gulubova M., Ananiev J., Yovchev Y., Julianov A., Karashmalakov A., Vlaykova T. (2013). The Density of Macrophages in Colorectal Cancer Is Inversely Correlated to TGF-Β1 Expression and Patients’ Survival. J. Mol. Hist..

[16] Zhou Q., Peng R.-Q., Wu X.-J., Xia Q., Hou J.-H., Ding Y., Zhou Q.-M., Zhang X., Pang Z.-Z., Wan D.-S. (2010). The Density of Macrophages in the Invasive Front Is Inversely Correlated to Liver Metastasis in Colon Cancer. J. Transl. Med..

[17] Cui Y.-L., Li H.-K., Zhou H.-Y., Zhang T., Li Q. (2013). Correlations of Tumor-Associated Macrophage Subtypes with Liver Metastases of Colorectal Cancer. Asian Pac. J. Cancer Prev. APJCP.

[18] Hamm A., Prenen H., Van Delm W., Di Matteo M., Wenes M., Delamarre E., Schmidt T., Weitz J., Sarmiento R., Dezi A. (2016). Tumour-Educated Circulating Monocytes Are Powerful Candidate Biomarkers for Diagnosis and Disease Follow-up of Colorectal Cancer. Gut.

[19] Franklin R.A., Liao W., Sarkar A., Kim M.V., Bivona M.R., Liu K., Pamer E.G., Li M.O. (2014). The Cellular and Molecular Origin of Tumor-Associated Macrophages. Science.

[20] Väyrynen J.P., Haruki K., Lau M.C., Väyrynen S.A., Zhong R., Dias Costa A., Borowsky J., Zhao M., Fujiyoshi K., Arima K. (2021). The Prognostic Role of Macrophage Polarization in the Colorectal Cancer Microenvironment. Cancer Immunol. Res..

[21] Pinto M.L., Rios E., Durães C., Ribeiro R., Machado J.C., Mantovani A., Barbosa M.A., Carneiro F., Oliveira M.J. (2019). The Two Faces of Tumor-Associated Macrophages and Their Clinical Significance in Colorectal Cancer. Front. Immunol..

[22] Accolla R.S., Tosi G. (2012). Optimal MHC-II-Restricted Tumor Antigen Presentation to CD4+ T Helper Cells: The Key Issue for Development of Anti-Tumor Vaccines. J. Transl. Med..

[23] Reeves E., James E. (2017). Antigen Processing and Immune Regulation in the Response to Tumours. Immunology.

[24] Bandola-Simon J., Roche P.A. (2019). Dysfunction of Antigen Processing and Presentation by Dendritic Cells in Cancer. Mol. Immunol..

[25] Warabi M., Kitagawa M., Hirokawa K. (2000). Loss of MHC Class II Expression Is Associated with a Decrease of Tumor-Infiltrating T Cells and an Increase of Metastatic Potential of Colorectal Cancer: Immunohistological and Histopathological Analyses as Compared with Normal Colonic Mucosa and Adenomas. Pathol. Res. Pract..

[26] Pagès F., Mlecnik B., Marliot F., Bindea G., Ou F.-S., Bifulco C., Lugli A., Zlobec I., Rau T.T., Berger M.D. (2018). International Validation of the Consensus Immunoscore for the Classification of Colon Cancer: A Prognostic and Accuracy Study. Lancet.

[27] Matsushita K., Takenouchi T., Shimada H., Tomonaga T., Hayashi H., Shioya A., Komatsu A., Matsubara H., Ochiai T. (2006). Strong HLA-DR Antigen Expression on Cancer Cells Relates to Better Prognosis of Colorectal Cancer Patients: Possible Involvement of c-Myc Suppression by Interferon-Gammain Situ. Cancer Sci..

[28] Andersen S., Rognum T., Lund E., Meling G., Hauge S. (1993). Strong HLA-DR Expression in Large Bowel Carcinomas Is Associated with Good Prognosis. Br. J. Cancer.

[29] D’Angelo E., Natarajan D., Sensi F., Ajayi O., Fassan M., Mammano E., Pilati P., Pavan P., Bresolin S., Preziosi M. (2020). Patient-Derived Scaffolds of Colorectal Cancer Metastases as an Organotypic 3D Model of the Liver Metastatic Microenvironment. Cancers.

[30] Kuang D.-M., Wu Y., Chen N., Cheng J., Zhuang S.-M., Zheng L. (2007). Tumor-Derived Hyaluronan Induces Formation of Immunosuppressive Macrophages through Transient Early Activation of Monocytes. Blood.

[31] D’Elios M.M., Vallese F., Capitani N., Benagiano M., Bernardini M.L., Rossi M., Rossi G.P., Ferrari M., Baldari C.T., Zanotti G. (2017). The Helicobacter Cinaedi Antigen CAIP Participates in Atherosclerotic Inflammation by Promoting the Differentiation of Macrophages in Foam Cells. Sci. Rep..

[32] Chistiakov D.A., Killingsworth M.C., Myasoedova V.A., Orekhov A.N., Bobryshev Y.V. (2017). CD68/Macrosialin: Not Just a Histochemical Marker. Lab. Investig..

[33] Pagliari M., Munari F., Toffoletto M., Lonardi S., Chemello F., Codolo G., Millino C., DELLA Bella C., Pacchioni B., Vermi W. (2017). Helicobacter pylori Affects the Antigen Presentation Activity of Macrophages Modulating the Expression of the Immune Receptor CD300E through miR-4270. Front. Immunol..

[34] D’Elios M.M., Josien R., Manghetti M., Amedei A., De Carli M., Cuturi M.C., Blancho G., Buzelin F., Del Prete G., Soulillou J.P. (1997). Predominant Th1 Cell Infiltration in Acute Rejection Episodes of Human Kidney Grafts. Kidney Int..

[35] Erreni M., Mantovani A., Allavena P. (2011). Tumor-Associated Macrophages (TAM) and Inflammation in Colorectal Cancer. Cancer Microenviron. Off. J. Int. Cancer Microenviron. Soc..

[36] Zhang Y., Rajput A., Jin N., Wang J. (2020). Mechanisms of Immunosuppression in Colorectal Cancer. Cancers.

[37] Lu P., Weaver V.M., Werb Z. (2012). The Extracellular Matrix: A Dynamic Niche in Cancer Progression. J. Cell Biol..

[38] Walker C., Mojares E., del Río Hernández A. (2018). Role of Extracellular Matrix in Development and Cancer Progression. IJMS.

[39] Song J.J., Ott H.C. (2011). Organ Engineering Based on Decellularized Matrix Scaffolds. Trends Mol. Med..

[40] Hoshiba T., Tanaka M. (2013). Breast Cancer Cell Behaviors on Staged Tumorigenesis-Mimicking Matrices Derived from Tumor Cells at Various Malignant Stages. Biochem. Biophys. Res. Commun..

[41] Piccoli M., D’Angelo E., Crotti S., Sensi F., Urbani L., Maghin E., Burns A., De Coppi P., Fassan M., Rugge M. (2018). Decellularized Colorectal Cancer Matrix as Bioactive Microenvironment for in Vitro 3D Cancer Research. J. Cell. Physiol..

[42] Sensi F., D’Angelo E., Piccoli M., Pavan P., Mastrotto F., Caliceti P., Biccari A., Corallo D., Urbani L., Fassan M. (2020). Recellularized Colorectal Cancer Patient-Derived Scaffolds as In Vitro Pre-Clinical 3D Model for Drug Screening. Cancers.

[43] Pinto M.L., Rios E., Silva A.C., Neves S.C., Caires H.R., Pinto A.T., Durães C., Carvalho F.A., Cardoso A.P., Santos N.C. (2017). Decellularized Human Colorectal Cancer Matrices Polarize Macrophages towards an Anti-Inflammatory Phenotype Promoting Cancer Cell Invasion via CCL18. Biomaterials.

[44] Boss J.M. (1997). Regulation of Transcription of MHC Class II Genes. Curr. Opin. Immunol..

[45] Reith W., LeibundGut-Landmann S., Waldburger J.-M. (2005). Regulation of MHC Class II Gene Expression by the Class II Transactivator. Nat. Rev. Immunol..

[46] Holling T.M., Schooten E., Langerak A.W., van den Elsen P.J. (2004). Regulation of MHC Class II Expression in Human T-Cell Malignancies. Blood.

[47] Murphy S.P., Tomasi T.B. (1998). Absence of MHC Class II Antigen Expression in Trophoblast Cells Results from a Lack of Class II Transactivator (CIITA) Gene Expression. Mol. Reprod. Dev..

[48] Morimoto Y., Toyota M., Satoh A., Murai M., Mita H., Suzuki H., Takamura Y., Ikeda H., Ishida T., Sato N. (2004). Inactivation of Class II Transactivator by DNA Methylation and Histone Deacetylation Associated with Absence of HLA-DR Induction by Interferon-γ in Haematopoietic Tumour Cells. Br. J. Cancer.

[49] Fabbri M., Croce C.M., Calin G.A. (2008). MicroRNAs. Cancer J..

[50] Codolo G., Toffoletto M., Chemello F., Coletta S., Soler Teixidor G., Battaggia G., Munari G., Fassan M., Cagnin S., de Bernard M. (2020). Helicobacter Pylori Dampens HLA-II Expression on Macrophages via the Up-Regulation of MiRNAs Targeting CIITA. Front. Immunol..

[51] Yoon S.O., Kim E.K., Lee M., Jung W.Y., Lee H., Kang Y., Jang Y.-J., Hong S.W., Choi S.H., Yang W.I. (2016). *NOVA1* Inhibition by MiR-146b-5p in the Remnant Tissue Microenvironment Defines Occult Residual Disease after Gastric Cancer Removal. Oncotarget.

[52] Deng X., Wu B., Xiao K., Kang J., Xie J., Zhang X., Fan Y. (2015). MiR-146b-5p Promotes Metastasis and Induces Epithelial-Mesenchymal Transition in Thyroid Cancer by Targeting ZNRF3. Cell Physiol. Biochem..

[53] Zhu Y., Wu G., Yan W., Zhan H., Sun P. (2017). MiR-146b-5p Regulates Cell Growth, Invasion, and Metabolism by Targeting PDHB in Colorectal Cancer. Am. J. Cancer Res..

[54] Zhang P., Ma Y., Wang F., Yang J., Liu Z., Peng J., Qin H. (2011). Comprehensive gene and microRNA expression profiling reveals the crucial role of hsa-let-7i and its target genes in colorectal cancer metastasis. Mol. Biol. Rep..

[55] Baer C., Squadrito M.L., Laoui D., Thompson D., Hansen S.K., Kiialainen A., Hoves S., Ries C.H., Ooi C.-H., De Palma M. (2016). Suppression of microRNA activity amplifies IFN-γ-induced macrophage activation and promotes anti-tumour immunity. Nature.

[56] Kryczek I., Zou L., Rodriguez P., Zhu G., Wei S., Mottram P., Brumlik M., Cheng P., Curiel T., Myers L. (2006). B7-H4 Expression Identifies a Novel Suppressive Macrophage Population in Human Ovarian Carcinoma. J. Exp. Med..

[57] Noy R., Pollard J.W. (2014). Tumor-Associated Macrophages: From Mechanisms to Therapy. Immunity.

[58] da Cunha B.R., Domingos C., Stefanini A.C.B., Henrique T., Polachini G.M., Castelo-Branco P., Tajara E.H. (2019). Cellular Interactions in the Tumor Microenvironment: The Role of Secretome. J. Cancer.

[59] Ardighieri L., Missale F., Bugatti M., Gatta L.B., Pezzali I., Monti M., Gottardi S., Zanotti L., Bignotti E., Ravaggi A. (2021). Infiltration by CXCL10 Secreting Macrophages Is Associated With Antitumor Immunity and Response to Therapy in Ovarian Cancer Subtypes. Front. Immunol..

[60] Biswas S.K., Mantovani A. (2010). Macrophage Plasticity and Interaction with Lymphocyte Subsets: Cancer as a Paradigm. Nat. Immunol..

[61] Knüpfer H., Preiss R. (2010). Serum Interleukin-6 Levels in Colorectal Cancer Patients—a Summary of Published Results. Int. J. Colorectal. Dis..

[62] Denning T.L., Wang Y., Patel S.R., Williams I.R., Pulendran B. (2007). Lamina Propria Macrophages and Dendritic Cells Differentially Induce Regulatory and Interleukin 17–Producing T Cell Responses. Nat. Immunol..

[63] Orsini G., Legitimo A., Failli A., Ferrari P., Nicolini A., Spisni R., Miccoli P., Consolini R. (2013). Defective Generation and Maturation of Dendritic Cells from Monocytes in Colorectal Cancer Patients during the Course of Disease. IJMS.

[64] García-Lora A., Algarra I., Collado A., Garrido F. (2003). Tumour Immunology, Vaccination and Escape Strategies: HLA and the Tumour Immune Escape. Eur. J. Immunogenet..

[65] Hsu S.-D., Chu C.-H., Tsou A.-P., Chen S.-J., Chen H.-C., Hsu P.W.-C., Wong Y.-H., Chen Y.-H., Chen G.-H., Huang H.-D. (2007). MiRNAMap 2.0: Genomic Maps of MicroRNAs in Metazoan Genomes. Nucleic Acids Res..

[66] Monslow J., Govindaraju P., Puré E. (2015). Hyaluronan—A Functional and Structural Sweet Spot in the Tissue Microenvironment. Front. Immunol..

[67] Schwertfeger K.L., Cowman M.K., Telmer P.G., Turley E.A., McCarthy J.B. (2015). Hyaluronan, Inflammation, and Breast Cancer Progression. Front. Immunol..

[68] Kim H., Cha J., Jang M., Kim P. (2019). Hyaluronic Acid-Based Extracellular Matrix Triggers Spontaneous M2-like Polarity of Monocyte/Macrophage. Biomater. Sci..

[69] Chanmee T., Ontong P., Itano N. (2016). Hyaluronan: A Modulator of the Tumor Microenvironment. Cancer Lett..

[70] Sato N., Kohi S., Hirata K., Goggins M. (2016). Role of Hyaluronan in Pancreatic Cancer Biology and Therapy: Once Again in the Spotlight. Cancer Sci..

[71] Ropponen K., Tammi M., Parkkinen J., Eskelinen M., Tammi R., Lipponen P., Agren U., Alhava E., Kosma V.M. (1998). Tumor Cell-Associated Hyaluronan as an Unfavorable Prognostic Factor in Colorectal Cancer. Cancer Res..

[72] Zhang G., Guo L., Yang C., Liu Y., He Y., Du Y., Wang W., Gao F. (2016). A Novel Role of Breast Cancer-Derived Hyaluronan on Inducement of M2-like Tumor-Associated Macrophages Formation. OncoImmunology.

