# Supplementary material for: Tumor Cells and the Extracellular Matrix Dictate the Pro-Tumoral Profile of Macrophages in CRC
Source: Cancers (Basel). 2021 Oct 16;13(20):5199. doi: 10.3390/cancers13205199 (PMC8533926; doi:10.3390/cancers13205199)
Supplement: Supplementary file 1 [file cancers-13-05199-s001.zip › cancers-1420638-supplementary.pdf]

Figure S1

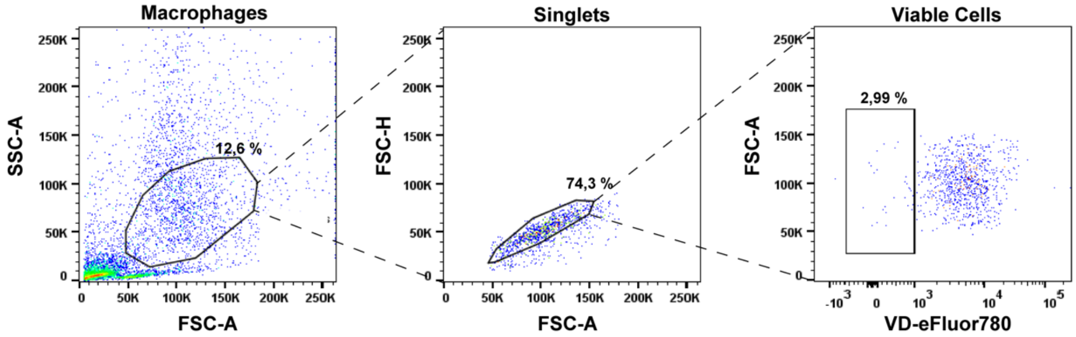

Figure S1. Representative cytograms of untreated monocytes. 2×10[6] monocytes from healthy donors were plated in 24-w plates and cultured in DMEM 10% human serum (HS), 4 mM Hepes, and 50 μg/mL gentamycin. After 5 d cells were harvested and analyzed by flow cytometry. Monocytes-differentiated macrophages population was first gated based on the FSC-A and SSC-A; then, single cells were gated based on FSC-A and FSC-H and dead cells were excluded using the fixable cell viability dye (VD) eFluor780. The population of viable cells is insufficient to proceed with the analysis of CD14 and CD68 expression.

Figure S2

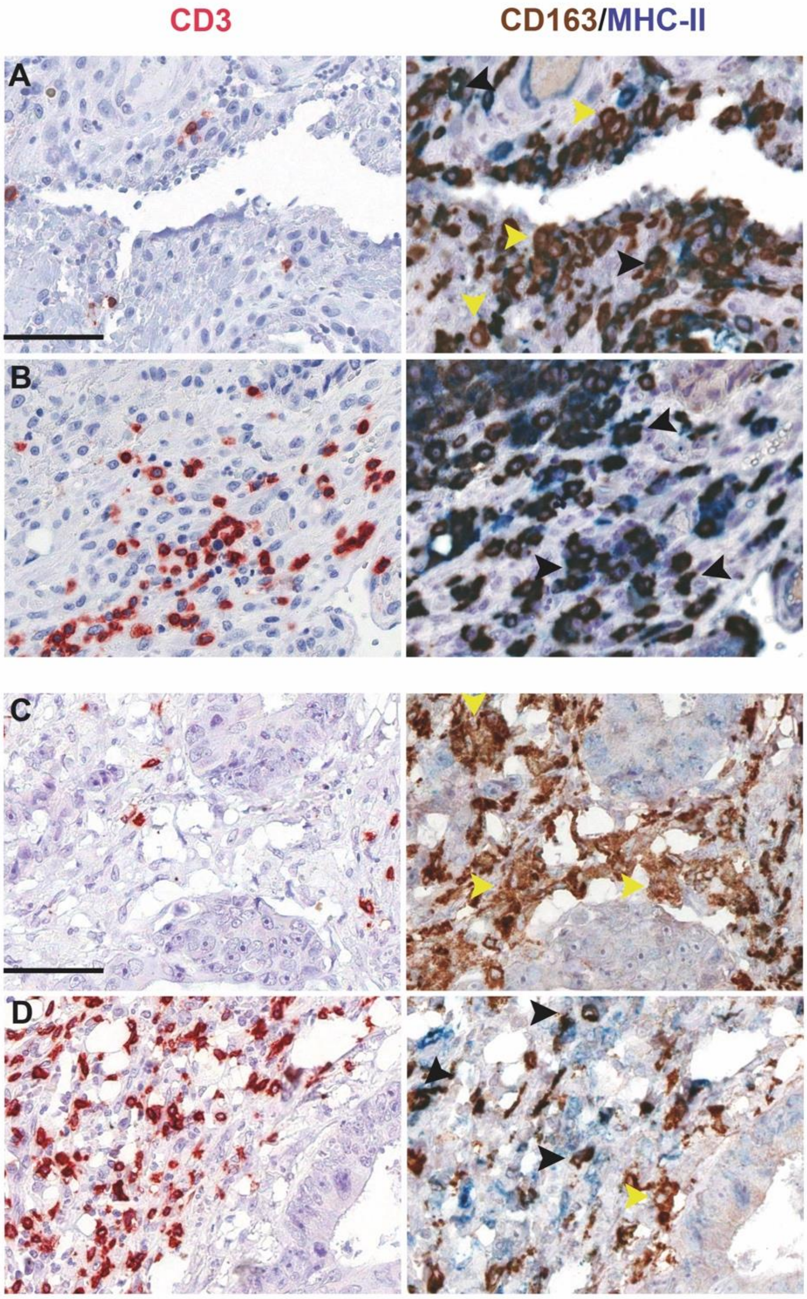

Figure S2. A higher number of MHC-II$^{dim/-}$ CD163^{+}macrophages correlate with a lower number of CD3^{+} T cells infiltrating tumor areas in CRC. Sequential immunostainings for CD3, MHC-II and CD163 were performed in CRC cases (n=8). CD3 was developed in Aminoethilcarbazole (AEC) whereas MHC-II in Ferangi Blue and CD163 in DAB (as labelled). Two representative CRC cases (A and B single case, C and D single case) show areas enriched in CD163+ MHC-II$^{dim/-}$ macrophages (yellow arrows) characterized by a low density of CD3^{+} T cells, and areas enriched in CD163^{+} MHC-II^{+}

macrophages (black arrows) with numerous CD3+ T cells. Images were taken as snapshot from digitalized slides at 400x of magnification, scale bar 66 mm.

Figure S3

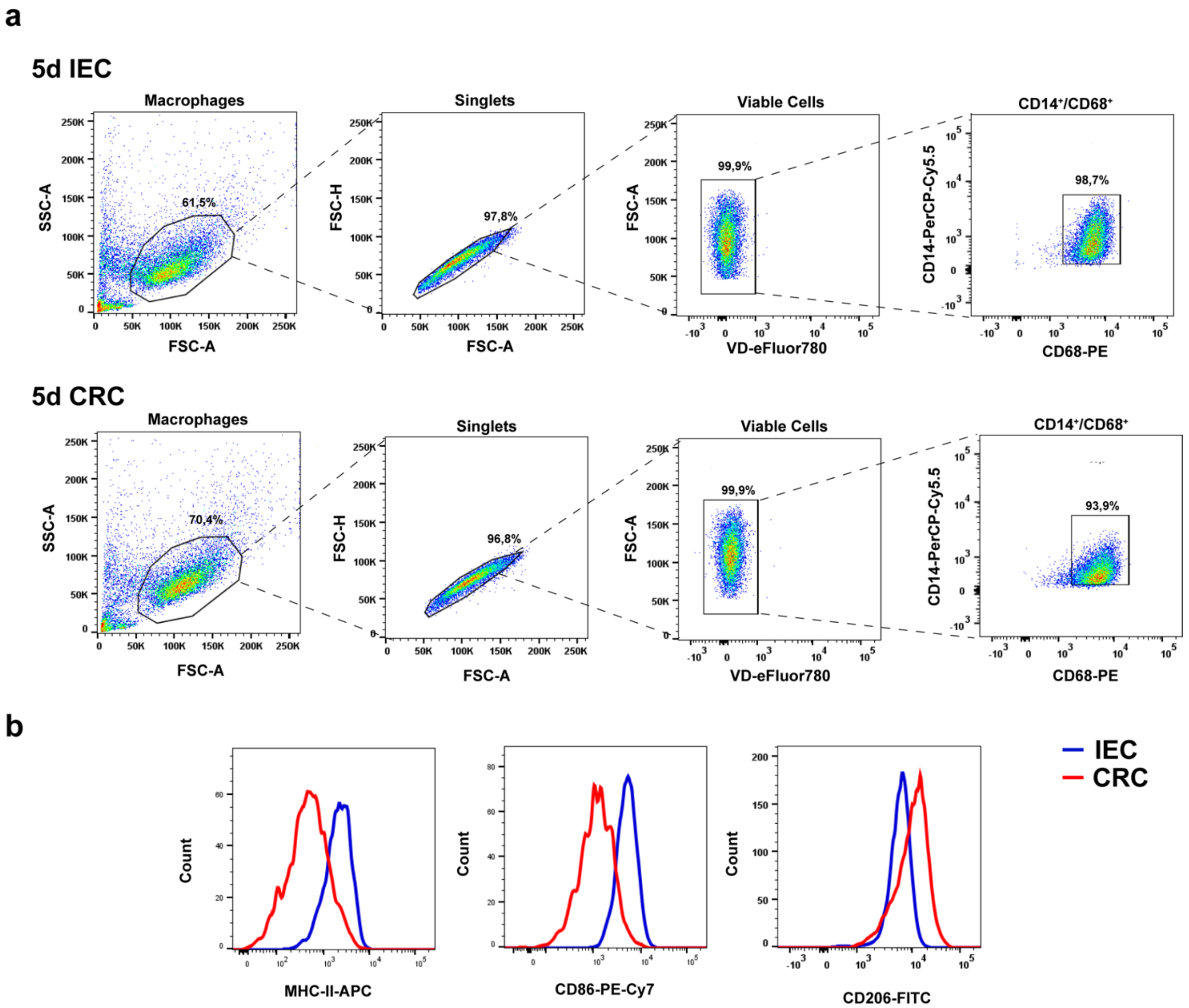

Figure S3. Examples of the flow cytometric gating strategy adopted for data analysis. Monocytes from healthy donors were plated on the bottom chamber of a transwell system and co-cultured with normal human intestinal epithelial cells (IEC) or colon cancer cells (CRC) plated onto the upper chamber. (a) After 5d co-culture, monocytes differentiated into macrophages in both conditions, IEC (middle panel) and CRC (lower panel), as demonstrated by the bigger morphology of the cells and by the expression of CD14/CD68 with respect to T_0 (upper panel), cells were harvested from the bottom chamber and analyzed by flow cytometry according to the following gating strategy. Monocytes-differentiated macrophages population was first gated based on the FSC-A and SSC-A, then, single cells were gated based on FSC-A and FSC-H and dead cells were excluded using the fixable cell viability dye (VD) eFluor780. Macrophages were further gated based on CD14 and CD68 expression. (b) The subpopulations of CD14+/CD68+ live cells were analyzed for the expression of MHC-II, CD86 and CD206 as indicated in the representative histograms. The expression of these markers has been compared between macrophages co-cultured with IEC (blu) and CRC (red). The same gating strategy was used in all the experiments aimed at defining monocytes/macrophages profile.

Figure S4

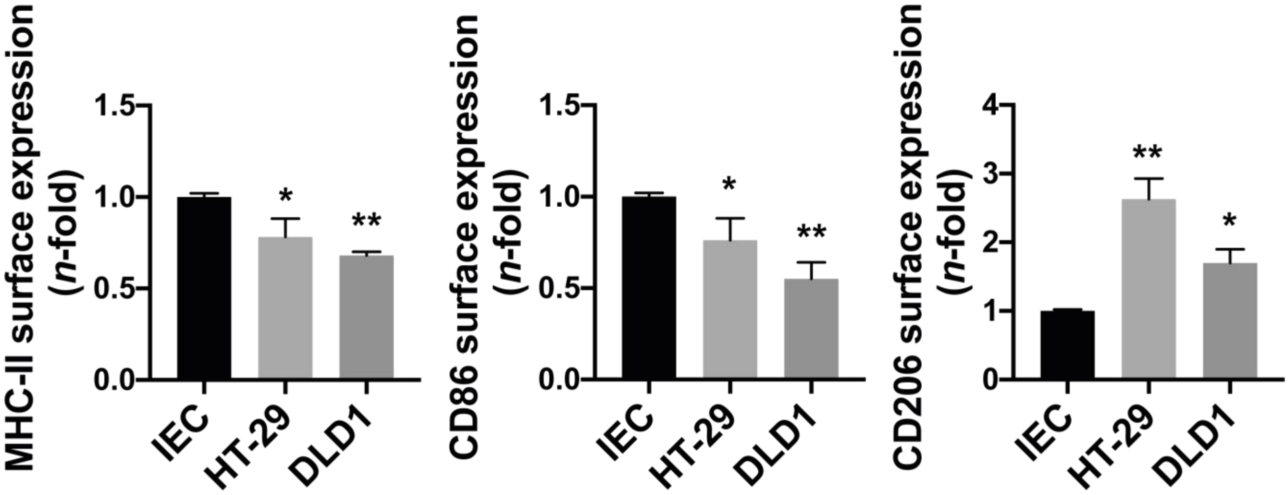

Figure S4. Profile of monocyte-differentiated macrophages co-cultured with tumor cells. Monocytes from healthy donors were plated on the bottom chamber of a transwell system and co-cultured with normal human intestinal epithelial cells (IEC) or HT-29 and DLD1 colon cancer cells plated onto the upper chamber. After 5 d cells were harvested from the bottom chamber and analyzed by flow cytometry for the expression of MHC-II, CD86, and CD206 gated on $CD68^+$ cells. Data are expressed as *n*-fold vs. IEC ± SEM of seven independent experiments from seven different donors. Significance was determined by Student's *t* test. *$p < 0.05$, **$p < 0.01$.

Figure S5

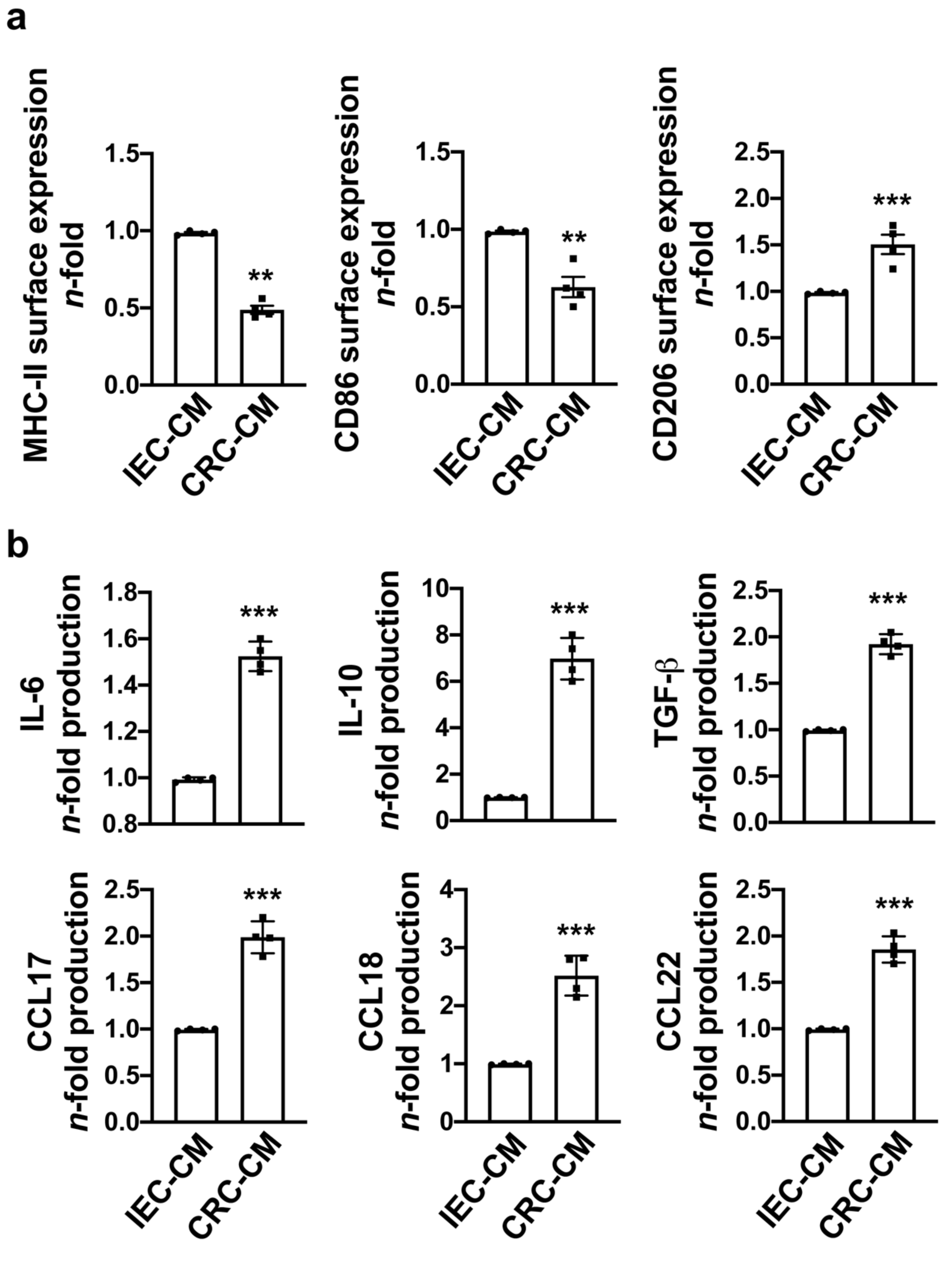
a
MHC-II surface expression
n-fold
1.5
1.0
0.5
0.0
**
IEC-CM
CRC-CM
CD86 surface expression
n-fold
**
CD206 surface expression
n-fold
2.5
2.0
***
b
IL-6
n-fold production
1.8
1.6
1.4
1.2
0.8
***
IL-10
n-fold production
10
8
6
4
2
0
TGF-β
n-fold production
CCL17
n-fold production
CCL18
n-fold production
3
1
CCL22
n-fold production

Figure S5. Conditioned medium from colorectal cancer cells promotes monocytes differentiation towards a pro-tumoral macrophages like-profile. (a) $2x10^6$ monocytes from healthy donors were plated in 24-w plate and stimulated with 15% of normal intestinal cells (IEC) or HCT116 (CRC) conditioned media (CM). After 5 d cells were harvested and analyzed by flow cytometry for the expression of MHC-II, CD86 and CD206 gated on $CD68^+$ cells. Data are expressed as *n*-fold vs IEC-CM, ± SEM of 4 independent experiments from 4 different donors. **(b)** Culture supernatants from the above cells were harvested and IL-6, IL-10, TGF-b, CCL17, CCL18, and CCL22 released were quantified by ELISA assay. Data are expressed as *n*-fold vs IEC-CM, ± SEM of 4 independent experiments from 4 different donors. Significance was determined by Student's *t*-test $^{**}p < 0.01$, $^{***}p < 0.001$.

Figure S6

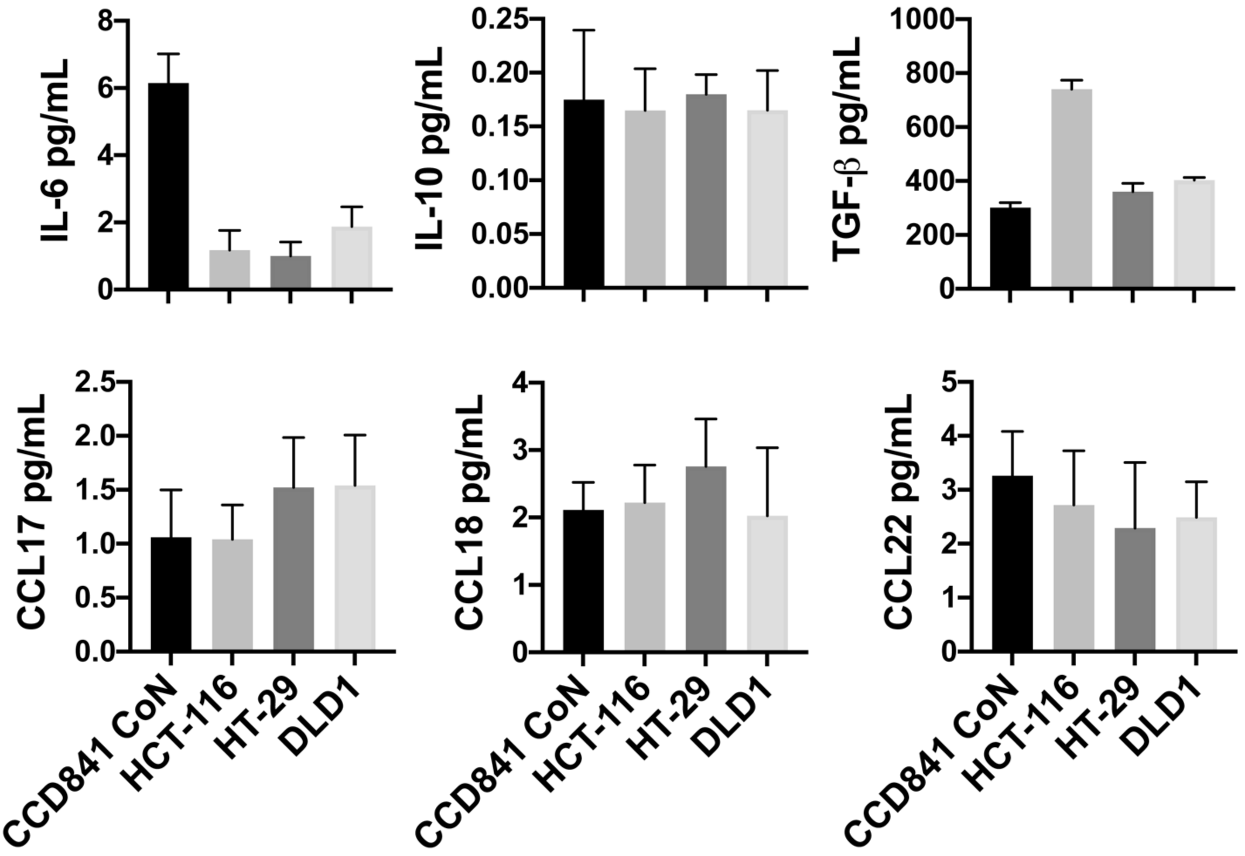

Figure S6. Cytokines and chemokines in conditioned media from colorectal cancer cells. Culture media from CCD841 CoN, HCT-116, HT-29 and DLD1 cells were harvested and IL-6, IL-10, TGF-b, CCL17, CCL18, and CCL22 released were quantified by ELISA assay. Data are expressed as pg/mL.

Figure S7

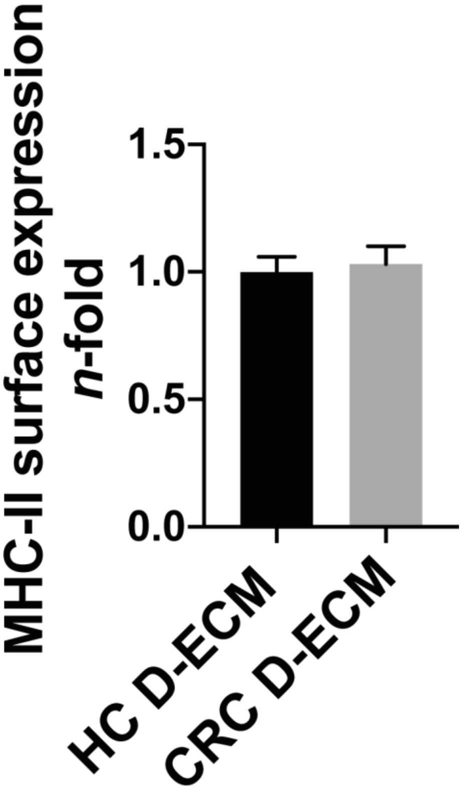

Figure S7. Conditioned media from D-ECM does not affect MHC-II surface expression. Normal decellularized matrix (HC D-ECM) and matched tumor decellularized matrix (CRC D-ECM) were maintained in DMEM 10% HS for 3 d. D-ECM conditioned media were then applied to monocytes cultures for 3 d. Cells were harvested and analyzed by flow cytometry for the expression of MHC-II gated on $CD14^+/CD68^+$ cells. Data are expressed as *n*-fold *vs* Normal D-ECM of each patient ± SD on 4 patients.

Figure S8

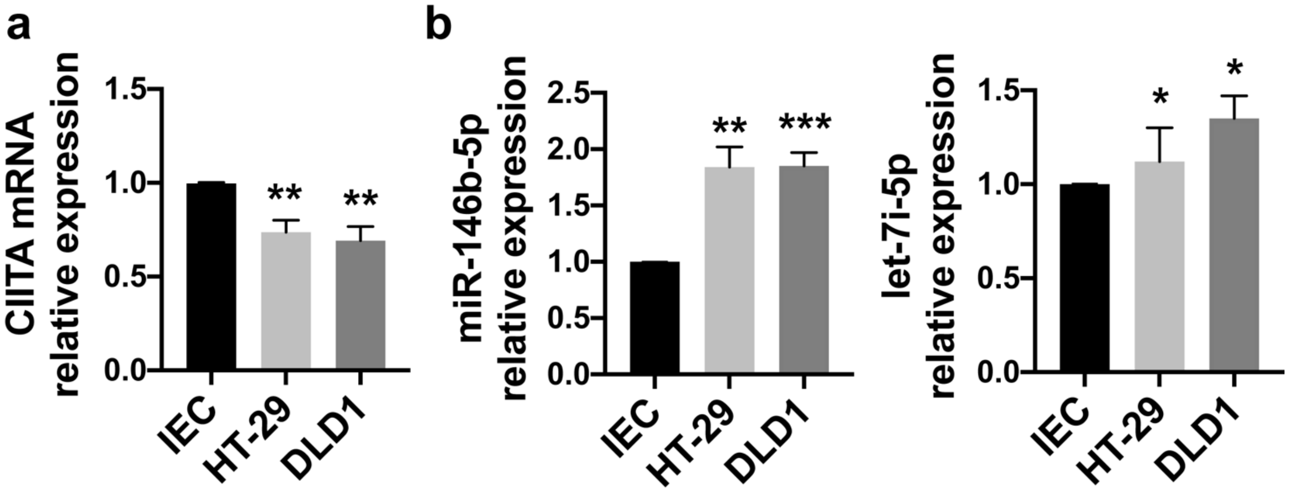

Figure S8. Expression of CIITA, miR-146b-5p and let-7i-5p in monocyte-derived macrophages. Monocytes from healthy donors were seeded on the bottom chamber of a transwell system and co-cultured with normal human intestinal epithelial cells (IEC; control cells) or HT-29 and DLD1 colon cancer cells seeded onto the upper chamber. After 5 d, cells were harvested from the bottom chamber and the expression of CIITA mRNA was analyzed by qRT-PCR. Data were normalized to the endogenous reference gene β-actin (left panel). The relative expression of miR-146b-5p and let-7i-5p were evaluated and data were normalized to an endogenous reference gene (U6). Values of macrophages cultured with control cells were taken as the reference and set as 1; the expression of macrophages cultured with CRC cells was relative to the expression of control cells (right panel). Significance was determined by Student's *t* test. *$p<0.05$ and ***$p<0.001$.

Figure S9

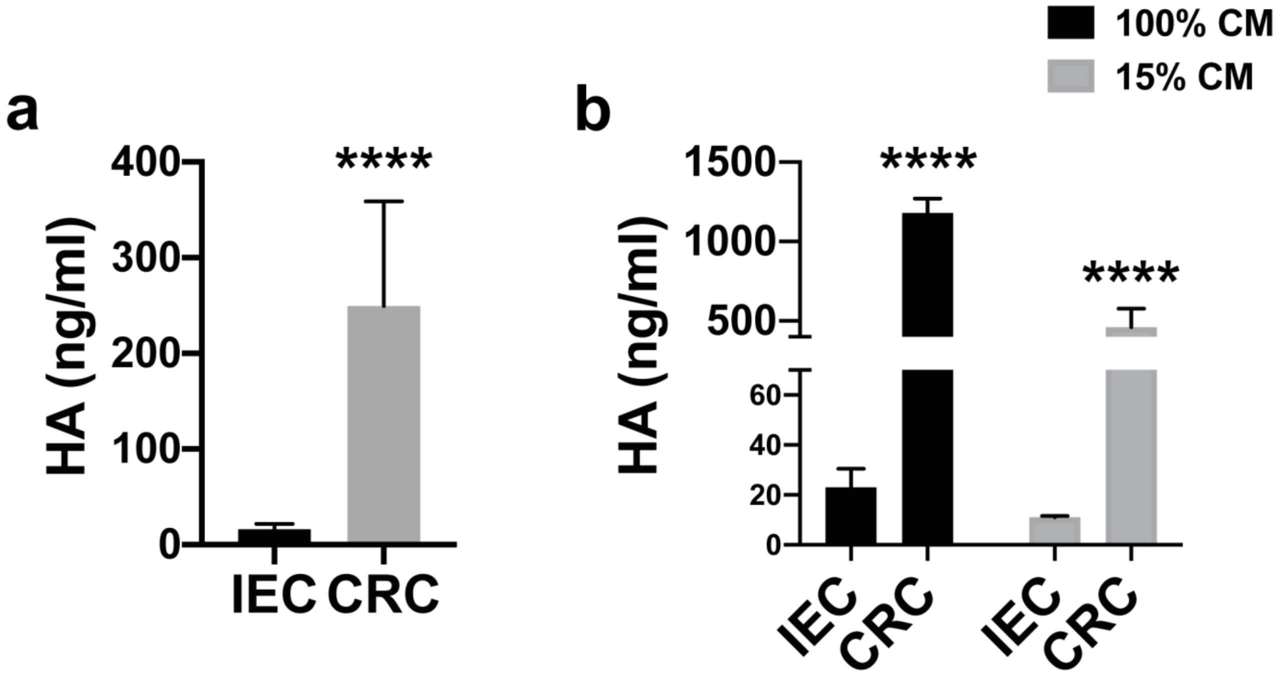

Figure S9. HA quantification in monocytes co-cultured with tumor cells and in HCT116 conditioned medium. (a) Monocytes from healthy donors were plated on the bottom chamber of the transwell system and co-cultured with normal intestinal cells (IEC) or HCT116 (CRC) plated onto the upper chamber. After 5 d, culture supernatants were harvested and HA in culture supernatants was quantified by ELISA assay. Data are expressed as ng/ml, ± SEM of 7 independent experiments from 7 different donors. **(b)** HA was quantified in normal intestinal cells (IEC) and HCT116 (CRC) conditioned media 100% and diluted to the 15% (concentration adopted to stimulate monocytes). Significance was determined by Student's t-test and two-way ANOVA ****$p < 0.0001$.

Supplementary Table SI

		Median Fluorescence Intensity (MFI)		
		**MHC-II**	**CD86**	**CD206**
**IEC (CCD841 CoN)**	Donor 1	6418	2588	1621
	Donor 2	12027	6892	3259
	Donor 3	5018	4010	2616
	Donor 4	2111	4575	2558
	Donor 5	5521	4228	2781
	Donor 6	6334	5577	5992
	Donor 7	9751	2132	1636
**CRC (HCT-116)**	Donor 1	4364	2485	3566
	Donor 2	2165	1585	5540
	Donor 3	2560	922	5232
	Donor 4	1647	3065	4860
	Donor 5	4306	2114	4172
	Donor 6	4371	2677	12703
	Donor 7	5656	1322	2814
**IEC-CM (CCD841 CoN)**	Donor 1	13033	3561	3972
	Donor 2	10723	2568	4231
	Donor 3	5964	3028	5486
	Donor 4	7284	3052	5204
**CRC-CM (HCT-116)**	Donor 1	7310	2208	6792

	Donor 2	5254	1284	6851
	Donor 3	2629	1754	7957
	Donor 4	3350	2495	6453
**HC D-ECM**	Pt 1	3649	6054	1063
	Pt 2	5792	26206	2920
	Pt 3	5585	21167	4256
	Pt 4	4483	22190	3510
	Pt 5	1452	11108	1308
	Pt 6	8392	24104	4490
	Pt 7	7579	19185	3406
	Pt 8	10747	20715	3643
**CRC D-ECM**	Pt 1	2558	1065	1983
	Pt 2	4196	20282	5921
	Pt 3	3498	18110	5408
	Pt 4	2323	15030	4225
	Pt 5	1014	8736	1942
	Pt 6	5533	18941	6335
	Pt 7	3450	18633	11693
	Pt 8	6083	17862	6045

Flow cytometry Median Fluorescence Intensity (MFI) raw data referred to Figures 2, 4 and Supplementary Figure S3.

Supplementary Table SII

		**Cytokines and chemokines concentrations (pg/mL)**					
		**IL-6**	**IL-10**	**TGF-b**	**CCL-17**	**CCL-18**	**CCL-22**
**IEC (CCD841 CoN)**	Donor 1	4061,48	214,73	1065,29	398,32	252,75	223,82
	Donor 2	663,41	215,7	608,7	1449,56	1225,21	359,64
	Donor 3	966,51	66,93	527,38	137,6	437,57	380,29
	Donor 4	404,12	132,11	294,67	361,54	599,75	262,45
	Donor 5	187,06	53,97	908,36	377,68	191,15	156,03
	Donor 6	888,01	94,03	403,09	175,67	880,51	107,83
	Donor 7	284,98	44,34	1088,84	198	302,63	480,01
**CRC (HCT-116)**	Donor 1	7147,4	2104,38	2335,12	873,11	964,71	455,22
	Donor 2	966,51	2264,91	1350,1	3215,12	2424,27	969,9
	Donor 3	2815,84	736,32	1123,32	293,09	1225,21	907,11
	Donor 4	802,43	1585,34	804,46	987,01	1619,34	626,02
	Donor 5	379,54	464,17	2780,5	1156,1	539,8	359,2

	Donor 6	1801,7	1316,5	994,04	433,21	2060,4	263,07
	Donor 7	544,32	394,21	2776,55	489,06	805,01	1022,43
**IEC-CM (CCD841 CoN)**	Donor 1	566,17	384,28	1164,9	169,14	476,63	175,42
	Donor 2	386,29	168,21	998,89	329,17	395,09	463,78
	Donor 3	238,69	102,38	474,2	606,12	206,52	238,45
	Donor 4	806,76	109,87	619,99	117,22	696,35	471,3
**CRC-CM (HCT-116)**	Donor 1	877,56	2305,7	2213,4	334,89	1334,55	356,77
	Donor 2	562,78	1244,7	1788	655,04	908,7	834,8
	Donor 3	381,91	665,47	972,1	1078,9	444,01	450,67
	Donor 4	1202,07	878,99	1208,99	257,89	1966,4	801,21
**HC D-ECM**	Pt 1	275,66	433,98	1615,7	374,55	2023,33	238,2
	Pt 2	116,72	127,6	671,09	398,0	239,68	174,6
	Pt 3	154,42	242,9	1608,7	1076,23	1089,7	259,82
	Pt 4	655,07	465,57	679,77	585,3	285,93	538,83
	Pt 5	1077,96	481,09	1257,77	716,31	912,1	841,01
	Pt 6	854,1	1071,6	622,96	232,19	1216,09	778,66
	Pt 7	948,43	720	937,75	283,2	1166	589
	Pt 8	1188,88	503	1000,5	192,03	986,16	894
**CRC D-ECM**	Pt 1	485,1	512,1	1809,55	466,4	2968,63	359,15
	Pt 2	443,55	167,9	794,57	557	331,68	274,8
	Pt 3	224,97	399,09	2302,1	1540	1751,65	549,2
	Pt 4	3289	1528,9	933,32	780	718,67	1167
	Pt 5	3361,35	949,5	1544,44	1238	1056,26	982,2
	Pt 6	1322,06	1427,2	688,99	275	1455,51	979,05
	Pt 7	6021,67	1296	2008,66	354	1410,86	706,8
	Pt 8	4161,08	995,94	1350,62	265	1331,32	1117,5

Cytokines and chemokines concentration (pg/mL) detected in culture supernatants from monocytes co-culture with i) normal intestinal cells (CCD841 CoN) or CRC cells (HCT-116), ii) stimulated with conditioned media (CM) from normal intestinal cells (CCD841 CoN) or CRC cells (HCT-116) or iii) co-cultured with normal (HC) or tumor (CRC) decellularized matrices (D-ECM).
